# Osteoarthritis: Epidemiology, Pathogenesis, and Treatment

**DOI:** 10.1002/mco2.70727

**Published:** 2026-04-09

**Authors:** Tianrui Chen, Wenlong Chen, Tianpeng Xu, Hong Wang, Yuheng Zhang, Li Wang, Sijie Zhu, Huaqiang Tao, Xing Yang

**Affiliations:** ^1^ Orthopedics and Sports Medicine Center Suzhou Municipal Hospital The Affiliated Suzhou Hospital of Nanjing Medical University Suzhou Jiangsu China; ^2^ Department of Anesthesiology Suzhou Municipal Hospital The Affiliated Suzhou Hospital of Nanjing Medical University Suzhou Jiangsu China; ^3^ Department of Stomatology Suzhou Municipal Hospital The Affiliated Suzhou Hospital of Nanjing Medical University Suzhou Jiangsu China; ^4^ Gusu School Nanjing Medical University Suzhou Jiangsu China

**Keywords:** epidemiology, epigenetic regulation, histone methylation, osteoarthritis, signaling pathways, therapy

## Abstract

Osteoarthritis (OA) is the most common chronic joint disorder and a major cause of disability worldwide. Once regarded as a consequence of cartilage wear, OA is now recognized as a complex whole‐joint disease involving coordinated pathological changes in articular cartilage, synovium, and subchondral bone. Disease progression is driven by chronic low‐grade inflammation, metabolic dysregulation, oxidative stress, and abnormal cellular responses to mechanical stress. These processes are mediated by interconnected signaling networks that regulate inflammatory responses, extracellular matrix (ECM) metabolism, and tissue remodeling. Epigenetic mechanisms, such as DNA methylation, histone modifications, and noncoding RNAs, are increasingly recognized as regulators of OA‐related gene expression. However, how signaling networks integrate with epigenetic regulation, particularly histone methylation, remains incompletely understood. In this review, we summarize the epidemiological burden and major risk factors of OA, describe pathological remodeling across joint tissues, and discuss key signaling pathways involved in OA pathogenesis before outlining epigenetic mechanisms. We also highlight the role of histone methylation in inflammation, metabolic imbalance, and tissue remodeling, and summarize current nonpharmacological, pharmacological, injectable, and surgical treatment strategies. Together, this review provides an integrated overview of the epidemiology, pathogenesis, and treatment of OA.

## Graphical abstract

Osteoarthritis is a prevalent degenerative joint disease with a growing global burden. Its development involves complex interactions among epidemiological factors, pathological changes, molecular signaling, and epigenetic regulation. Understanding these mechanisms provides insights into disease progression and supports the development of therapeutic strategies, including nonpharmacological interventions, pharmacologic treatments, regenerative medicine, and surgical management.

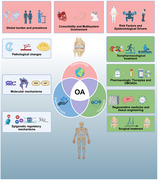

## 1 Introduction

Osteoarthritis (OA) is a chronic condition due to ongoing structural changes in joints, resulting in a gradual loss of ability to participate in activities over an extended time period. The initiation and progression of OA occur through a complex interrelated series of pathological mechanisms involving degeneration of cartilage, inflammation of synovial tissue, abnormal remodeling of subchondral bone, and persistent disruption of homeostasis within the joint's microenvironment [1]. The diversity of joint compartments and their structural and functional characteristics renders OA a multifactorial and highly heterogeneous disease, making it unlikely that a single mechanism can fully explain its progression [2].

The development of OA at the molecular level involves continual stimulation of inflammatory responses, metabolic reprogramming, increased oxidative stress, and disruptions in cellular fate‐related homeostasis [3, 4]. These elements are interconnected through multiple cellular signaling pathways that are coactivated or dynamically interact to regulate cellular homeostasis and the adaptive capacity of joint tissues [5, 6]. Research suggests that aberrantly activated or suppressed canonical signaling pathways in OA‐associated cells under pathological conditions are linked to imbalances in extracellular matrix (ECM) synthesis and degradation, dysregulated expression of proinflammatory mediators, and pathological remodeling of joint tissues. However, how the functions of these pathways are hierarchically organized or change over time has not yet been fully integrated or explained [7].

Beyond signaling pathways themselves, long‐term cellular responses to mechanical stimuli, inflammatory microenvironments, and metabolic states depend on higher‐order regulatory mechanisms governing gene expression. Multiple epigenetic mechanisms—including DNA methylation, posttranslational histone modifications, and noncoding RNAs (ncRNAs)—have been demonstrated to participate in the regulation of OA‐related pathological processes [8]. Histone methylation is increasingly becoming of interest as one of the many types of epigenetic modifications due to its highly specific and reversible regulation, and potential to develop new therapies for disease. Since changes in the level of histone methylation can affect the regulation of transcriptional activity of genes that are important in signaling pathways (e.g., inflammatory, differentiation, and remodeling), further understanding of how histone methylation interacts with other cellular events involved in joint degeneration and how it regulates the onset and progression of OA is needed [9].

Most OA therapies today focus on pain relief and functional improvement but do little to change the course of the disease [10]. As researchers learn more about how OA develops and how it changes at the cellular level, they are exploring different types of treatments that may modify the pathology of OA; examples include targeting inflammation, abnormal signaling pathways, metabolic dysregulation, and tissue remodeling [11]. While these attempts offer additional opportunities beyond conventional symptom‐based treatments, they will still require more general testing and extended assessment to be ultimately applied in the clinic.

In this context, this review summarizes the epidemiological and etiologic drivers of OA, outlines pathological alterations across joint tissues, and then describes the key signaling pathways and network crosstalk that regulate inflammation, metabolism, and tissue remodeling. Then, we will integrate the epigenetic regulatory landscape of OA, focusing on the mechanistic evidence and interventional potential of histone methylation. Finally, we will summarize the current clinical evidence, recent progress, and clinical hurdles in nonpharmacological interventions, injectable therapies, surgical interventions, and disease‐modifying OA drugs (DMOADs), and aim to provide a conceptual framework for subsequent mechanistic studies and precision intervention strategies.

## 2 Epidemiology of OA

In recent decades, OA has evolved from being viewed primarily as an age‐related degenerative disorder to a complex chronic condition with substantial global health and socioeconomic implications. Understanding its epidemiology therefore requires consideration not only of the overall disease burden and prevalence patterns, but also of the underlying drivers that shape its distribution across populations. In this section, we summarize the global burden of OA and its prevalence trends, and discuss the major risk factors and epidemiological determinants that contribute to its increasing incidence. We also consider the frequent coexistence of OA with multiple systemic conditions, highlighting the broader multisystem context in which the disease develops.

### 2.1 Global Burden and Prevalence

OA is one of the most prevalent chronic joint diseases worldwide, and the epidemiological burden of OA has been increasing for at least the past three decades. The most recent estimates from the 2021 Global Burden of Disease study reported approximately 607 million people affected by OA globally in 2021, a 136% increase over the 1990 estimate. In the same period, incident cases reached 466 million, and OA accounted for an estimated 213 million disability‐adjusted life years (DALYs) [12]. From 1990 to 2021, the age‐standardized incidence and prevalence of OA increased at a rate of about 0.3–0.4% per annum, with this increase further accelerated by population aging, the increasing prevalence of obesity, and changes in lifestyle [13]. The global prevalence of OA is estimated at about 7.6%, and the total number of people affected by OA may approach 1 billion by 2050, with OA being one of the leading causes of disability among people aged 70 years and older [14].

By joint site, knee OA comprises the majority of the OA phenotype and contributes the most DALYs in total (although the burden from OA of the hip and fingers continues to rise) [15]. By 2050, there will be 74.9 and 78.6% more OA of the knee and hip, 48.6% more OA of the fingers, and 95.1% more OA of other joints compared with today [16]. OA is more common among women than men, and these differences widen progressively with age. The increased burden of disease in women likely relates to declining levels of estrogen in the postmenopausal state, increased immune reactivity in women, and sex‐specific genomic effects on bone metabolism [17].

Shifts in age structure are also of importance. Across all countries, the incidence of OA follows an inverted U‐shaped distribution with age, peaking in the 50–64‐year age group. Although the overall incidence peaks in this age group, the fastest growth in incidence has recently been observed among younger adults aged 30–44 years [18]. With the growing prevalence of metabolic disorders and obesity, the incidence of OA among individuals younger than 50 years has increased by more than 25% compared with previous reports [19]. This observation indicates a shift in the epidemiological profile of OA, moving from a disease primarily associated with aging toward a systemic chronic condition. Loss of productivity in younger patients due to OA accounts for almost 60% of the entire economic burden attributed to OA [20].

There are marked gradients globally across the socio‐demographic index (SDI) for OA—countries at the higher end have age‐standardized prevalence rates that are higher than in other settings due to higher expected age at diagnosis and better diagnostic capacity. Middle‐SDI countries and above also have a higher number of cases in aggregate due to population size and obesity prevalence [19]. East and South Asia account for over 35% of OA cases globally, with the largest increases occurring in the Middle East and Latin America [16]. The numbers in China, India, and Indonesia have each risen by over 50% since 1990, driven by population aging, urbanization, and lifestyle changes [21]. Growth in case numbers has markedly slowed in high‐SDI countries since then, but the absolute burden has still increased due to demographic aging; in contrast, increases in middle‐SDI countries and above are largely due to changes in population structure and higher prevalence of metabolic disorders [22].

Health inequities associated with OA are becoming increasingly evident. Low‐income countries face a dual burden of high prevalence and high disability as a result of limited healthcare access and delayed diagnosis [23]. OA has risen to the seventh leading cause of years lived with disability globally, ranking only behind low back pain and major depressive disorder. Compared with 1990, OA‐related DALYs have increased by approximately 47%, with more than 60% of the additional burden originating from Asia [24]. Collectively, these trends indicate that the epidemiological landscape of OA is undergoing a systemic transformation—characterized by sustained growth in case numbers, a shift toward younger age groups, widening sex‐ and region‐specific disparities, and escalating societal impact—positioning OA as one of the most challenging chronic disabling diseases worldwide.

### 2.2 Risk Factors and Epidemiological Drivers

The rising prevalence of OA, particularly among older adults, is attributable to the increasingly complex interplay of multiple factors, including population aging, obesity and metabolic dysregulation, mechanical strain and shear stress, sex‐ and hormone‐related influences, and genetic predisposition. Together, these factors contribute to the evolving epidemiological profile of OA, steadily transforming it from a local degenerative disease of joints into the chronic, systemic metabolic, and inflammatory condition we recognize today.

Aging is clearly one of the most powerful risk factors for OA. With increasing age, chondrocytes progressively acquire senescence‐associated features, including irreversible cell‐cycle arrest, and exhibit greater susceptibility to apoptosis and a reduced capacity for ECM synthesis [25]. These senescent cells secrete the senescence‐associated secretory phenotype (SASP) that characteristically includes inflammatory cytokines such as interleukin‐6 (IL‐6) and matrix metalloproteinases (MMPs), which activate further inflammatory and matrix‐degradative pathways and contribute to a chronic state of inflammaging [26, 27]. More recent work has shown that senescence signaling in cartilage and subchondral bone can integrate to amplify local inflammatory responses and tissue remodeling, further accelerating cartilage degeneration. Multiomics data indicate the accumulation of senescence‐associated differentially expressed genes that are significantly enriched in phosphoinositide 3‐kinase (PI3K)–AKT, mitogen‐activated protein kinase (MAPK), and p53 signaling pathways, and aberrant activation of these pathways is associated with immune cell infiltration, implicating a central role of the immune–inflammatory axis in OA pathogenesis [28]. This senescence‐mediated inflammatory landscape creates a permissive biological niche for OA development and aligns with the increasing prevalence of the disease with age.

Obesity and metabolic dysregulation have become the major and predominant causes of the increasing OA burden in recent decades. Aside from increased load‐bearing, metabolic dysregulation in obesity stimulates the secretion of adipokines and proinflammatory cytokines, including IL‐6 and tumor necrosis factor‐alpha (TNF‐α), causing systemic low‐grade inflammation and establishing a chronic state of metabolic inflammation [25]. Dysregulated metabolic signaling interferes with the homeostatic demands of various OA‐related cells such as chondrocytes, synoviocytes, and osteoclasts, forcing local tissues into a self‐sustaining chronic inflammatory and sclerotic state [29]. The effects of obesity are not only due to mechanical overload but are more intrinsically linked to systemic metabolic signals. Recent meta‐analyses show that body mass index (BMI) ≥24 kg/m^2^ carries a 30% higher risk for the progression of knee OA (odds ratio [OR] = 1.30, 95% confidence interval [CI] 1.09–1.56) [30]. Obesity can lead to low‐grade chronic inflammation and dysregulated secretion of adipokines, thereby potentiating cartilage matrix degradation and abnormal remodeling of subchondral bone. Notably, studies have revealed that the combination of obesity and reduced skeletal muscle mass—termed sarcopenic obesity—is associated with an even greater risk for OA (OR = 1.78, 95% CI 1.35–2.34) [31]. This association reflects the combined effects of metabolic dysfunction (e.g., insulin resistance, mitochondrial dysfunction, and chronic oxidative stress), which promote degenerative changes in cartilage and subchondral bone through inflammatory signaling and disruption of adenosine triphosphate (ATP) production. Overall, metabolic dysregulation rather than mechanical overload is suggested to be the key pathogenic mechanism underlying the association between obesity and the initiation and progression of OA.

Mechanical stress and occupational exposure constitute equally pivotal components within the epidemiologic framework of OA. Excessive loading over prolonged periods, repetitiveness, and fatigue from these loading postures may result in microdamage and stress concentrations in cartilage and subchondral bone, leading to inflammation and invasion of the synovial vasculature. The prevalence of early‐onset knee or hip OA is markedly higher in workers engaged in heavy manual labor and athletes than in the general population, largely due to joint injury, excessive mechanical loading, and postural stress [32, 33]. Some reports suggest that abnormal mechanical stress compromises mechanotransduction and the cellular energy metabolic state of chondrocytes, activating multiple forms of cell death (e.g., apoptosis and ferroptosis), as well as Ca^2^^+^ influx via mechanosensitive ion channels, thereby perpetuating inflammation and ECM degradation and providing a molecular–mechanical basis for cartilage degeneration [34]. Magnetic resonance imaging (MRI) and finite‐element modeling demonstrate that meniscal tibial extrusion causes a mismatch between bone and cartilage coverage in the tibiofemoral joint, leading to increased local contact stress. Each millimeter increase in medial meniscal extrusion is associated with an approximately 11–13% higher risk of radiographic OA progression over the subsequent 4 years. Thus, an imbalanced mechanical environment of the cartilage itself is considered a precursor to load‐induced structural damage [35]. Last, biomechanical studies show that female patients with knee OA exhibit greater knee valgus at maximum knee flexion and a lower hip adduction moment during maximal knee loading in stair descent; concomitantly, abnormal activity is observed in the quadriceps, hamstrings, and gastrocnemius. This pathological redistribution of load within the knee joint may contribute to the rapid progression of degenerative changes in the joint [36].

Sex‐ and endocrine‐related factors further exacerbate the heterogeneity of OA. Both the incidence and disability burden of OA are consistently higher in women than in men, with the disparity becoming particularly pronounced after menopause. Emerging evidence indicates that this sex difference cannot be fully explained by variations in joint anatomy or loading patterns, but is more closely associated with estrogen deficiency and adipokine‐related signaling dysregulation [37]. Declining estrogen levels disrupt cartilage metabolic homeostasis by modulating signaling pathways mediated by estrogen receptor α, estrogen receptor β, and G protein‐coupled estrogen receptor, thereby suppressing ECM synthesis while amplifying inflammatory responses and oxidative stress, ultimately accelerating subchondral bone remodeling [38, 39]. Concurrently, adipose tissue‐derived leptin and resistin levels are markedly elevated in women, promoting the expression of proinflammatory cytokines such as IL‐6 and TNF‐α, as well as MMPs including MMP1 and MMP3, thereby driving synovial inflammation and cartilage matrix degradation. In contrast, circulating levels of adiponectin and ghrelin are reduced, weakening their anti‐inflammatory and antiapoptotic effects and leading to a disrupted “hormone–metabolism–inflammation” equilibrium [40]. Moreover, in postmenopausal women, increased activity of bone marrow adipocytes and intensified crosstalk between adipokines and estrogen signaling further promote synovial angiogenesis and subchondral bone sclerosis, underscoring the pivotal role of endocrine‐metabolic dysregulation in the heightened susceptibility to and severity of OA in women [40, 41].

OA susceptibility is also influenced by genetic factors. Genome‐wide association studies (GWAS) have reported polymorphisms in genes such as growth differentiation factor 5 (GDF5) and SMAD family member 3 (SMAD3), which are associated with chondrocyte differentiation, ECM homeostasis, and bone remodeling [42, 43]. Similarly, the risk of developing OA is markedly increased by environmental exposures associated with air pollution, particulate matter with an aerodynamic diameter ≤2.5 µm (PM2.5) exposure, and sedentary lifestyles related to urbanization [44, 45]. These findings highlight the strong possibility that gene–environment interactions predispose individuals to disease through shared oxidative and inflammatory signaling pathways.

**FIGURE 1:**
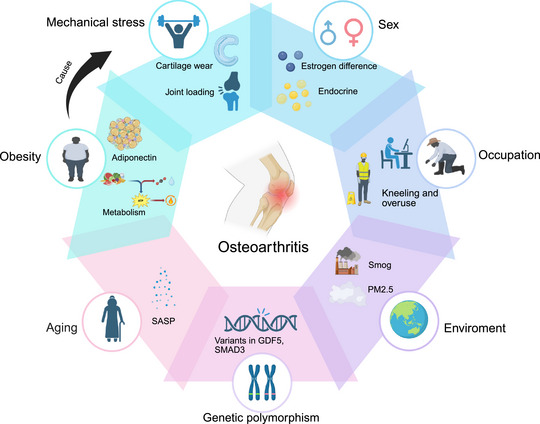
Major risk factors contributing to osteoarthritis development and progression. Mechanical, metabolic, genetic, occupational, and environmental factors collectively drive osteoarthritis pathogenesis. (Created with BioRender.com.)

Collectively, these epidemiological drivers of OA can be visualized as a tightly coupled “metabolic–mechanical–inflammatory” triad. Age furnishes a permissive background of frailty, increased body mass and metabolic inflammation provide sustained driving forces, mechanical stress and work‐related features determine anatomic distribution, while sex, genetic predisposition and environmental exposures converge to determine an individual's risk of disease trajectory and rate of progression (Figure 1).

### 2.3 Comorbidity and Multisystem Involvement

The presence of comorbidities in OA suggests that the disease is neither confined to the joint nor purely local, but rather represents a dynamic process shaped by local tissue alterations as well as distal systemic biological influences. The profile of comorbidities (metabolic, cardiovascular, chronic inflammatory, and dysregulated bone and muscle homeostasis) likely influences predisposition and manifestation of the disease and modulates pain generation, which structures are affected, and the efficacy of treatment. This is a prime source of heterogeneity in OA. The following section outlines the major links between comorbidities and multisystem phenomena in OA to set the stage for considering mechanism‐based stratification and targeted therapeutic pathways.

#### 2.3.1 Cardiovascular Diseases

Recent studies have increasingly revealed a close association between OA and cardiovascular dysfunction, suggesting that vascular pathology may constitute an important component of the systemic vulnerability underlying OA. A nationwide population‐based case–control study in Japan demonstrated that patients with knee OA exhibited a significantly higher overall risk of cardiovascular disease (CVD) compared with controls (OR = 1.19, 95% CI 1.12–1.27), with the risks of ischemic heart disease and stroke increased by approximately 18 and 22%, respectively, whereas no significant associations were observed for hip or hand OA [46]. This site‐specific phenomenon suggests that chronic inflammation and abnormal mechanical loading in weight‐bearing joints may promote vascular dysfunction through vascular stress responses.

OA and CVD are highly prevalent comorbid conditions in older populations, with hypertension and atherosclerosis being the most common manifestations. In the United States National Health and Nutrition Examination Survey (NHANES) study, the prevalence of peripheral arterial stenosis (≥50%) among individuals aged ≥70 years was 15%, while MRI‐based assessments indicated that the true prevalence may approach 28%, suggesting that the burden of peripheral atherosclerosis in OA populations may be underestimated [47]. Although bidirectional Mendelian randomization (MR) analyses based on NHANES 2024 and GWAS data did not identify a direct genetic causal relationship between OA and hypertension (OR = 1.02, 95% CI 0.93–1.13), their shared disease patterns support a systemic pathological basis involving metabolic–vascular–osteoarticular interactions [48]. Analysis of NHANES data from 1999 to 2018, including 48,372 adults, found that 48.0% had hypertension and 24.5% had OA, with 35.1% of patients having both conditions. OA was associated with an approximately 1.3‐fold increased risk of hypertension (OR = 1.32; 95% CI 1.16–1.50). This association was attenuated in individuals aged ≥60 years, with an OR of 1.08, but remained statistically significant [49]. Data from population‐based studies in Europe show that hypertension and OA are prevalent in over 60.7 and 55%, respectively, of individuals aged ≥70 years, highlighting their coexistence as two of the most common chronic diseases in aging populations that tend to cluster [50]. Further epidemiological studies indicate that patients with knee OA and comorbid hypertension have approximately 90% greater risks of radiographic progression and nearly 40% increased risk of symptomatic OA [51].

The previously described epidemiological link between OA and the process of vascular atherosclerosis is further supported by recent evidence from imaging and hemodynamic studies. In another study, OA patients showed greater carotid‐femoral pulse wave velocity, a measure of central arterial stiffness (9.40 ± 1.92 vs. 8.25 ± 1.26 m/s, *p* < 0.0001); however, there was no difference in femoral‐ankle pulse wave velocity. Multivariate regression models showed that central arterial stiffness was associated with knee OA after adjusting for age, blood pressure, lipids, BMI, and hs‐CRP, and was positively correlated with Kellgren–Lawrence grade (*r*_s_ = 0.23, *p* = 0.008) [52]. These findings suggest that central arterial compliance is considerably reduced in OA, with relative preservation of peripheral arterial function, indicating a central‐dominant pattern of vascular stiffening.

#### 2.3.2 Neurodegenerative Diseases

In recent years, accumulating evidence suggests a robust bidirectional association between OA and neurodegenerative diseases, referred to as the “brain–joint axis,” providing a conceptual framework in which OA patients exhibit a multidimensional disease process comprising dysregulation of the central nervous, immune, and endocrine systems [53]. A cross‐sectional analysis using data from the U.S. NHANES 2011–2020 (*n* = 11,117) showed a 95% higher risk of incident Parkinson's disease (PD) (OR = 1.95, 95% CI 1.44–2.62) among individuals with OA [54]. Beyond PD, the association between Alzheimer's disease (AD) and OA has also gained attention. A prospective cohort study using the UK Biobank provided further supporting evidence for a systemic link: during long‐term follow‐up of nearly 460,000 participants, OA was associated with a significantly higher risk of incident dementia (hazard ratio [HR] = 1.12, 95% CI 1.04–1.20), with the risk elevation particularly pronounced for AD (HR = 1.13, 95% CI 1.01–1.25). Additionally, OA patients also had thinner cortices and smaller surface areas in multiple other regions, specifically in the occipital, medial prefrontal cortex, and temporal areas [55]. More recently, MR analyses provided additional evidence for a causal role of OA in increasing the risk of AD (inverse‐variance weighted OR = 1.28, 95% CI 1.11–1.47) [56]. Importantly, OA and neurodegenerative diseases share broad epidemiological features: prevalence among the elderly, occurrence alongside chronic low‐grade inflammation and metabolic dysregulation, as well as shared lifestyle‐related risk factors [57]. These shared features provide further evidence for a common systemic mechanism underlying the existence of a “brain–joint axis.”

#### 2.3.3 Neuropsychiatric Disorders

Depression and anxiety are common in patients with OA and have recently been recognized as key determinants of outcome [58]. Multiple international epidemiological studies indicate that the pooled prevalence of depressive symptoms in individuals with OA is approximately 20% (95% CI 15.9–24.5%), highlighting a substantial psychological burden in this population. Among patients undergoing joint replacement surgery, this proportion increased from 5.1 to 15.4% between 2002 and 2012 [59]. Recent systematic reviews and meta‐analyses further show that the prevalence of depression among patients with knee OA is approximately 34% (95% CI 28–41%). Female sex, obesity, bilateral knee pain, higher Western Ontario and McMaster Universities Osteoarthritis Index (WOMAC) scores, slower walking speed, and unmarried or widowed status are all significantly associated with an increased risk of depression [60].

Pain intensity is positively correlated with the severity of depressive symptoms, and higher pain scores together with poorer joint function are often accompanied by worsening emotional disturbances. Regional epidemiological surveys have further revealed that approximately one‐third of patients with knee OA experience anxiety or depressive symptoms (anxiety 28.1%, depression 30.3%), with lower BMI, reduced quality of life, and insufficient social support identified as independent risk factors [61]. Social support scores are negatively associated with anxiety and depression, highlighting a close link between mental health and social connectedness. Emotional disorders and pain‐ and function‐related impairments interact bidirectionally, jointly exacerbating disease burden and diminishing quality of life. Emerging clinical evidence further suggests that anxiety and depression exert reciprocal effects on pain perception and therapeutic responsiveness in OA. In a prospective cohort study of 206 elderly hospitalized patients with knee OA, Yang and colleagues reported that 91% of patients exhibited varying degrees of depression and 31% had comorbid anxiety. Baseline levels of depression and anxiety were predictive of pain intensity at 3 months of follow‐up, indicating that negative emotional states significantly attenuate the analgesic efficacy of treatment [62].

#### 2.3.4 Gastrointestinal Diseases

The burden of gastrointestinal (GI) disorders in patients with OA has long been underestimated. Systematic reviews indicate that approximately 67% of individuals with OA have at least one chronic comorbidity, among which upper GI disorders represent one of the most common systemic comorbidities. Compared with non‐OA populations, patients with OA have an approximately twofold higher risk of developing peptic ulcer disease (prevalence ratio ≈ 2.36) [63]. This association is partly attributable to the older age profile and clustering of multiple comorbidities in OA populations; however, a more substantial driver is long‐term analgesic and anti‐inflammatory therapy. Widespread use of nonsteroidal anti‐inflammatory drugs (NSAIDs) and opioid analgesics among OA patients can precipitate a spectrum of GI toxicities throughout the GI tract, ranging from dyspepsia to peptic ulceration and GI bleeding. The risk is particularly pronounced in individuals who are older, have a history of ulcer disease, have concomitant cardiovascular conditions, or use anticoagulants concurrently. Accordingly, clinical guidelines generally classify OA populations as a high‐risk group requiring systematic assessment and individualized strategies for gastric mucosal protection [64].

Recent genetic epidemiological studies have also examined potential causal relationships between OA and GI diseases. MR analyses indicate a possible causal association between genetically predicted OA and gastroesophageal reflux disease (GERD) (OR ≈ 1.26). In contrast, no clear causal relationship has been identified for peptic ulcer disease or inflammatory bowel disease. Both univariable and multivariable MR analyses further suggest that the increased risk of GERD observed in individuals with OA may be partly mediated by opioid use, whereas NSAID use does not appear to play a significant mediating role [58]. Overall, these results raise the possibility that the comorbidity of OA with certain GI disorders may arise not only from shared risk factors and the toxicity of medications used for pain management, but also from more complex interactions involving pain management paradigms and regulation of the neuro–GI axis.

**FIGURE 2:**
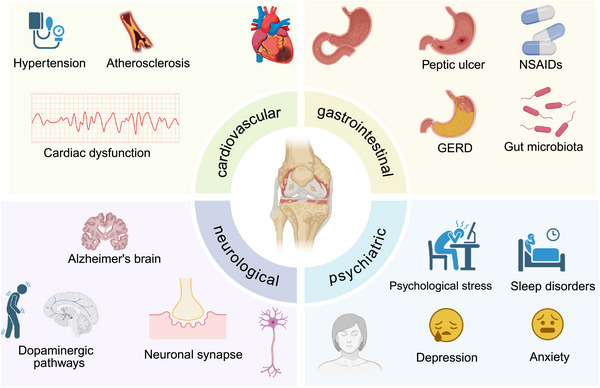
Osteoarthritis is associated with multisystem comorbidities. OA is linked to cardiovascular, gastrointestinal, neurological, and psychiatric disorders, highlighting the systemic nature of OA and its complex pathophysiological interactions. (Created with BioRender.com.)

In sum, OA can be considered a systemic rather than solely a joint disease, as multiorgan system comorbidities frequently occur alongside it. Epidemiological studies show that OA patients have a higher burden of CVDs, metabolic diseases, and GI complications, as well as neurodegenerative and mental health disorders, including depression and anxiety (Figure 2). These conditions coexist and interact with each other, often through common pathological mechanisms, including low‐grade inflammation, metabolic and immune dysfunction, and neuroendocrine dysregulation. Comorbidities contribute to exacerbating pain levels and functional impairment, making OA more difficult to manage, and influence treatment response, prognosis, and quality of life.

## 3 Pathological Changes in OA

The pathological changes seen in OA do not derive from a single tissue, nor are they simply points on a degenerative continuum; rather, OA is a derangement of the normal balance among multiple tissues within the joint—a dysfunction of cartilage, synovium, the underlying subchondral bone, the meniscus, and nearby periarticular tissues. Even the earliest changes associated with OA entail disruption of ECM homeostasis, the presence of low‐grade inflammation within the synovial membrane, and remodeling of the architecture of subchondral bone. These changes are temporally and spatially coupled to one another and propagate in self‐reinforcing loops through inflammatory signaling, mechanical stress, and cellular communication, leading to coordinated tissue dysfunction and failure. A whole‐joint understanding of OA behavior and opportunities for new treatment approaches is therefore essential for OA studies.

### 3.1 Articular Cartilage Degeneration

Articular cartilage degeneration is the central pathological hallmark of OA, and its progression involves multilevel alterations in tissue architecture and cellular behavior. Structural disruption is reflected by surface fibrillation, disorganization of the collagen network, and expansion of calcified cartilage zones (CCZs). Concurrently, chondrocytes undergo profound phenotypic changes, including enhanced ECM degradation, cellular senescence, and multiple forms of programmed cell death. These processes are accompanied by disturbances in the local microenvironment, such as hypoxia and oxidative stress, ultimately leading to the progressive loss of joint cushioning and lubricating functions.

Structurally, cartilage degeneration in OA occurs from the surface inward. The early event is irregularity and fibrillation of the surface, with slight swelling and increased water uptake. The damage progresses to fissured defects in the deeper zones, resulting in loss of cartilage material, formation of clefts, and local delamination of bearing surfaces [65]. With progressive degeneration, the CCZ is markedly thickened, and the tidemark duplicates and moves proximally. At the same time, blood vessels and sensory nerves invade from the subchondral bone into deeper zones of cartilage, disturbing the osteochondral interface [66]. Ultrastructural and histological studies reveal disorganization and rupture of collagen fibrils, increased porosity of the subchondral bone plate, and progressive formation of ridges and cysts [67]. Collectively, these structural changes compromise the transitional and shock‐absorbing capacity of the cartilage–bone interface, leading to shear stress concentration and accumulation of microdamage, thereby accelerating mechanical failure and the rapid, irreversible loss of cartilage function.

Overall, during OA progression, chondrocytes progressively lose their ability to maintain their homeostatic phenotype and acquire abnormal hypertrophic and dedifferentiated cellular phenotypes. These aberrant phenotypes drive the breakdown of the cartilage matrix and calcification, as hypertrophic chondrocytes—characterized by enlarged cell volumes and increased expression of osteogenic factors including collagen Type X alpha 1 chain (COL10A1), Runt‐related transcription factor 2 (RUNX2), MMP13, and vascular endothelial growth factor (VEGF)—promote matrix degradation and calcification [68]. Rim et al. demonstrated that decreased Type II collagen and proteoglycans are colocalized and together facilitate a phenotypic transition in which cartilage progressively loses its elastic characteristics [69]. Lian et al. further proposed that the loss of Type II collagen (COL2A1) activates the bone morphogenetic protein (BMP)–SMAD1 signaling pathway, promoting chondrocyte hypertrophy and ossification, whereas exogenous COL2A1 can partially reverse this process [70]. In parallel, a subset of chondrocytes undergoes dedifferentiation, characterized by upregulated Type I collagen expression and increased fibrotic activity, further reducing the elastic and mechanical buffering capacity of cartilage. Ultimately, under persistent inflammatory and catabolic stress, chondrocytes enter states of cellular senescence and dysregulated autophagy, predisposing cartilage to further degeneration.

**FIGURE 3:**
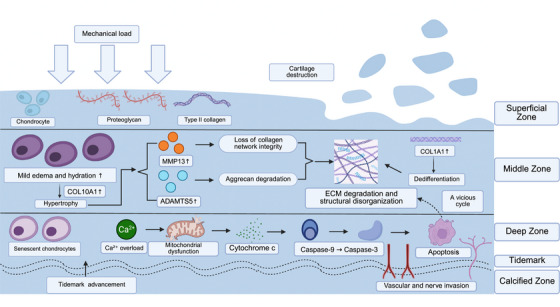
Cartilage degeneration during osteoarthritis progression. Mechanical overload disrupts cartilage homeostasis and promotes ECM breakdown, chondrocyte dysfunction, and apoptosis, driving progressive structural deterioration of articular cartilage. (Created with BioRender.com.)

Chondrocyte senescence is a stage during OA at which initially reversible cartilage injury becomes hardwired, leading to irreversible structural changes in cartilage. Senescent chondrocytes exhibit increased expression of p16, p21, and p53 and show sustained activation of the SASP. This phenotype promotes enhanced secretion of proinflammatory and matrix‐degrading mediators such as IL‐6 and MMP13, creating an increasingly prodegenerative microenvironment [71]. Continued cellular damage activates multiple programmed cell death pathways linked to mitochondrial dysfunction and endoplasmic reticulum (ER) stress, some of which trigger the caspase‐9/caspase‐3 cascade. Inflammatory signaling can also induce apoptosis through the Fas/FasL and TNF‐α/TNF receptor 1 (TNFR1) pathways [72]. The large‐scale loss of viable chondrocytes, together with proteolytic enzymes released from dying cells, further impairs the synthesis of Type II collagen and proteoglycans, thereby destabilizing the ECM and mechanical integrity of cartilage. These events form a vicious cycle known as “senescence–inflammation–apoptosis,” which further accelerates cartilage degeneration and worsens OA (Figure 3).

In OA, joint remodeling features a similar synergy between inflammation and pathological remodeling. Synovial activation, ECM stiffening, local changes in pH, and reduced oxygen tension combine to impose significant perturbations on tissue homeostasis. Aberrant invasion of blood vessels and sensory nerves from the subchondral bone introduces inflammatory cells and growth factors into the cartilage layer, breaching the native avascular microenvironmental barrier. Such combined and coordinated remodeling enables the localized degenerative process of OA to evolve into a more systemic pathological state [73, 74].

### 3.2 Subchondral Bone Remodeling

Subchondral bone is one of the first tissues to exhibit structural changes in OA and is subject to pathological remodeling throughout the disease. In the early stages, an increased rate of bone resorption by osteoclasts leads to rarefaction of the trabeculae and enlargement of marrow spaces, thinning of the subchondral plate, and increased porosity [75]. During this process, the tidemark is also compromised, allowing inflammatory mediators (as well as blood vessels and nerves) to invade the osteochondral interface, thereby breaching the joint barrier. Bone marrow lesions result from neovascularization and fibrosis, as well as engorgement of adipocytes and general disarray of marrow microarchitecture, indicating disruption of local metabolic and mechanostat homeostasis [67, 76]. As the disease evolves, bone formation responses in this environment become overdriven and produce features such as trabecular densification, subchondral bone sclerosis, and the presence of osteophytes. While a sclerotic thickening of the subchondral plate may appear beneficial due to increased load‐bearing capacity, it does so at the cost of reduced elasticity in load response and lower energy absorption per cycle, owing to increased frictional transfer of loads across other regions of the joint, thus worsening cartilage injury and microcrack accumulation [77]. In some areas, occlusion of the marrow cavity, failure to repair microfractures, and upward advancement of the zone of calcified cartilage at the osteochondral interface indicate the transition from compensatory remodeling to maladaptive reorganization [78].

Overall, pathobiological changes in subchondral bone follow a pattern of early resorption followed by later sclerosis accompanied by angiogenesis, which compromises the mechanical buffering capacity and metabolic support of the joint. Through the formation of a vascularized yet unevenly distributed scaffold and cytokine‐mediated signaling, these changes also induce intrinsic abnormalities in the overlying cartilage, thereby promoting localized degeneration and contributing to whole‐joint disease progression in OA.

### 3.3 Synovium–Fat Pad Interaction

The synovium and infrapatellar fat pad (IPFP) effectively belong to the same continuum and represent an inflammatory and metabolic hub within the joint cavity. In OA, fat pad hypertrophy, adipocyte hypertrophy, and focal necrosis are coupled with substantial immune cell infiltration, including macrophages and T lymphocytes. These cells release high levels of proinflammatory mediators, IL‐6, IL‐1β, and TNF‐α, which drive synovial fibroblast activation, angiogenesis, and ECM degradation, thereby sustaining synovial inflammation [79]. Concurrently, adipokines secreted by the fat pad actively participate in local disease processes. Leptin and resistin further amplify proinflammatory cytokine expression and promote immune cell infiltration [79]. Although adiponectin is traditionally considered anti‐inflammatory, it is frequently upregulated in OA‐associated fat pads and has been linked to cartilage degradation and angiogenesis in certain contexts [79, 80].

With disease progression, the structural boundary between the synovium and the IPFP becomes increasingly blurred, accompanied by aberrant growth of blood vessels and nerve fibers across both tissues. VEGF released from the IPFP promotes neovascularization into the synovium and subchondral bone, thereby exacerbating local exudation and pain responses [81]. Multiomics and functional studies further indicate activation of complement‐related molecules and pathways within the IPFP, suggesting a potential role as a local source of complement‐mediated inflammatory amplification, although the specific upregulation and function of C3 and C5 require further validation [82, 83]. At the metabolic level, IPFP‐derived fatty acid‐binding protein 4 and lysophosphatidylcholines are closely associated with oxidative stress and lipid metabolic dysregulation, contributing to adipocyte death and persistent inflammation [83]. While the combined effects of inflammatory and metabolic factors induce villous hyperplasia, fibrosis, and angiogenesis of the synovium, the fat pad also undergoes remodeling and becomes depleted of adipocytes, leading to the formation of a “fat pad–synovitis” pathological axis [84]. This cross‐tissue chronic inflammatory response impairs immune homeostasis in the joint cavity and stabilizes pathological signals in cartilage and subchondral bone through vascular and neural mechanisms, allowing OA progression and pain sensitization.

### 3.4 Vascular and Neural Remodeling

Compensatory resorption–repair dynamics differ significantly between early‐stage and late‐stage OA subchondral bone; in late‐stage OA, abnormal angiogenesis and sensory nerve ingrowth extend into deeper regions of the subchondral bone. Imaging and histological studies show a marked increase in vascular density in areas of trabecular remodeling, with some neovessels extending from the subchondral bone into the calcified cartilage and even the articular cartilage layer, creating conduits that allow inflammatory cells, cytokines, and metabolic mediators to access an interface that is otherwise relatively immune‐privileged [74, 85]. Preclinical evidence indicates that aberrantly activated preosteoclasts secrete high levels of platelet‐derived growth factor‐BB (PDGF‐BB), which promotes subchondral endothelial proliferation and angiogenesis and is tightly coupled to pathological bone formation; blockade of PDGF‐BB or its receptor signaling attenuates subchondral angiogenesis–bone remodeling and delays OA structural damage [86, 87]. In addition, VEGF and angiopoietin/angiopoietin‐like family factors, including angiopoietin‐like protein 2, are upregulated in OA cartilage and synovium and have been shown to disrupt local antiangiogenic barriers, thereby facilitating vascular invasion into subchondral bone and aggravating trabecular sclerosis and cartilage degeneration [73, 88].

Concomitant with vascular remodeling, pronounced sensory nerve remodeling occurs in the subchondral bone and at the osteochondral interface in OA. Animal models and human specimens consistently demonstrate a marked increase in calcitonin gene‐related peptide (CGRP)‐ and substance P‐positive sensory nerve fibers within OA subchondral bone, with a subset of fibers accompanying newly formed blood vessels into the CCZ and establishing close interactions with osteoblasts, osteoclasts, and immune cells [89, 90]. Mechanistic research reveals that netrin‐1, released from osteoclasts, promotes direct sensory axon guidance into subchondral bone. Complementary data from OA models show that either suppression of osteoclast activity or neutralization of netrin‐1 inhibits sensory nerve ingrowth into subchondral bone and reduces pain hypersensitivity characteristic of OA [91]. In parallel, aberrantly activated subchondral osteoblasts upregulate cyclooxygenase‐2 (COX‐2) and prostaglandin E_2_ (PGE_2_), which activates sensory neurons via the PGE_2_ receptor 4 (EP4), thereby sustaining nociceptive signaling and further perturbing bone remodeling. Genetic deletion of COX‐2 in osteoblasts or EP4 in sensory neurons decreases pain behaviors and joint structural damage within the same OA models [92, 93]. The nerve growth factor (NGF)–tropomyosin receptor kinase A axis amplifies this process, wherein NGF expression in subchondral bone and synovium is temporally associated with sensory nerve invasion and pain sensitization in OA models [94].

Aberrant angiogenesis and sensory nerve remodeling reinforce each other, establishing a self‐sustaining vascular–neural–inflammatory–pain pathogenic circuit that further aggravates subchondral sclerosis and synovitis, thereby driving disease progression and persistent pain and providing a mechanistic explanation for the transition of OA from a mechanically driven disorder to a chronic inflammatory disease.

## 4 Molecular Mechanisms of OA

**FIGURE 4:**
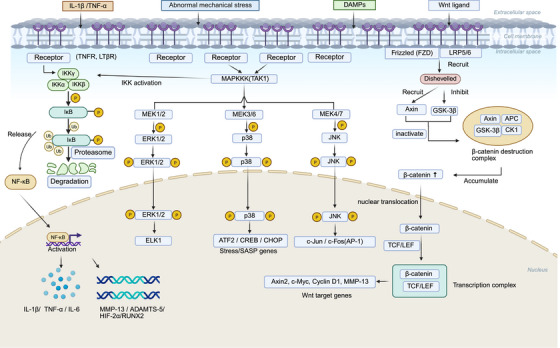
Signaling pathways driving osteoarthritis pathogenesis. Inflammatory stimuli, abnormal mechanical stress, and damage‐associated molecular patterns activate intracellular signaling cascades that promote chondrocyte inflammation, ECM degradation, and disease progression. (Created with BioRender.com.)

In OA, convergent signaling pathways create a web of connections between inflammation, metabolism, stress responses, and phenotypic changes. Inflammatory and mechanical signals activate the nuclear factor kappa B (NF‐κB) and MAPK pathways, driving production of matrix‐degrading MMPs and a disintegrin and metalloproteinase with thrombospondin motifs (ADAMTS), while Wnt/β‐catenin signaling regulates chondrocyte fate and osteochondral remodeling, together orchestrating the structural evolution of OA (Figure 4). The PI3K–AKT–mammalian target of rapamycin (mTOR) pathway regulates energy metabolism, autophagy, and cell survival. These convergent pathways act through overlapping transcriptional programs, oxidative stress, and metabolic states to propagate pathology from focal cartilage damage to joint‐wide involvement of the synovium, subchondral bone, and IPFP. Defining their coordination and amplification is key for mechanistic understanding and rational multitarget interventions.

### 4.1 NF‐κB Pathway

NF‐κB is a transcription factor complex that serves as a central regulator of inflammatory and catabolic gene expression in OA [95]. Proinflammatory cytokines, mechanical stress, and damage‐associated molecular patterns (DAMPs) activate the canonical NF‐κB pathway via the IκB kinase (IKK) complex, leading to phosphorylation and degradation of inhibitor of κBα (IκBα) and subsequent nuclear translocation of the p65/p50 heterodimer [6]. Activated NF‐κB drives the transcription of matrix‐degrading enzymes, including MMP1, MMP3, MMP13, and ADAMTS family proteases, as well as proinflammatory mediators such as COX‐2, inducible nitric oxide synthase (iNOS), PGE_2_, NO, IL‐6, and IL‐8 [96]. In addition, NF‐κB upregulates transcription factors such as hypoxia‐inducible factor 2 alpha (HIF‐2α) and RUNX2 in chondrocytes, thereby promoting terminal differentiation (hypertrophy) and aberrant ossification [97, 98].

NF‐κB exhibits extensive crosstalk with other signaling pathways in OA. For example, signal transducer and activator of transcription 3 (STAT3), a downstream effector of the Janus kinase/signal transducer and activator of transcription (JAK/STAT) pathway, cooperates with NF‐κB to amplify inflammatory gene expression. Activation of JAK/STAT3 signaling promotes NF‐κB p65 nuclear translocation and induces the expression of receptor activator of nuclear factor κB ligand (RANKL), MMP3, and MMP9 in cartilage, thereby exacerbating joint destruction [99, 100]. Conversely, NF‐κB activation can enhance STAT3 transcriptional activity, forming a positive feedback loop that jointly amplifies inflammation and matrix degradation [99]. Collectively, persistent NF‐κB activity—amplified via crosstalk with STAT3 and other pathways—contributes significantly to the chronic inflammation and cartilage degradation associated with OA and thus represents an attractive therapeutic target.

Studies have recently repositioned NF‐κB as one of the key molecules through which inflammation, aging, and matrix catabolism intersect in OA. In chondrocytes, continuous stimulation with IL‐1β induces nuclear translocation of p65/p50 and upregulation of catabolic and inflammatory mediators, including COX‐2, iNOS, MMP13, and ADAMTS‐5. Natural small molecules such as indole‐3‐propionic acid (IPA), orientin, and isoquercetin repress cartilage inflammation, apoptosis, and ECM degeneration in OA mice through inhibition of NF‐κB signaling—mainly by preventing IκBα phosphorylation/degradation and p65 nuclear translocation [101, 102, 103].

NF‐κB signaling is also relevant for the synovial and immune compartments beyond chondrocytes. Neuregulin 4 inhibits the polarization of synovial macrophages toward the proinflammatory M1 phenotype through the ErbB4–Stat5b–NF‐κB axis, reducing synovitis and cartilage damage, and further emphasizing the centrality of NF‐κB in the multitissue inflammatory OA network [104].

Despite being a key controller of inflammatory amplification, NF‐κB activation alone may be insufficient to drive OA initiation without accompanying metabolic dysfunction, suggesting that it primarily functions as a signal integrator rather than an independent disease trigger.

### 4.2 MAPK Signaling

The MAPK family, comprising extracellular signal‐regulated kinase (ERK), c‐Jun N‐terminal kinase (JNK), and p38 pathways, plays a central role in cellular stress and inflammatory responses. In OA, proinflammatory stimuli such as IL‐1β and TNF‐α concurrently activate MAPK signaling, with distinct functions among the subtypes. ERK primarily regulates chondrocyte proliferation and phenotypic modulation, whereas JNK and p38 are more closely associated with inflammation and apoptosis [105]. Activated JNK/p38 signaling induces activator protein‐1 transcription factors, thereby upregulating matrix‐degrading enzymes (e.g., MMP1 and MMP13) and inflammatory mediators, ultimately driving cartilage matrix breakdown and synovial inflammation [105].

In recent years, studies of MAPK signaling in OA cartilage have expanded beyond its traditional proinflammatory and matrix‐degradative roles to encompass regulation of cell fate, metabolic stress, and mechanotransduction. Picroside II has been shown to markedly suppress chondrocyte pyroptosis in the murine destabilization of the medial meniscus (DMM) model by inhibiting MAPK/NF‐κB signaling and attenuating NLR family pyrin domain containing 3 (NLRP3) inflammasome activation, thereby slowing cartilage degeneration [106]. Sun reported that long ncRNA (lncRNA) SNHG7 is significantly downregulated in OA cartilage and IL‐1β‐stimulated chondrocytes; through the SNHG7/miR‐324‐3p/DUSP1 axis, SNHG7 suppresses p38 MAPK phosphorylation, reduces TNF‐α and IL‐6 production, and limits chondrocyte apoptosis, suggesting its potential as an upstream modulator of p38‐mediated inflammatory responses [107]. Beyond inflammation, MAPK signaling is increasingly linked to cellular senescence and mitochondrial homeostasis. Recent studies demonstrate that overexpression of the mitochondrial kinase phosphatase and tensin homolog (PTEN)‐induced kinase 1 (PINK1) restores autophagy/mitophagy in chondrocytes, downregulates p38 MAPK/NF‐κB activation, reduces ROS accumulation and SA‐β‐gal‐positive cells, and significantly alleviates OA progression both in vivo and in vitro. These findings position the p38 MAPK–NF‐κB axis as a critical link connecting inflammation, mitochondrial dysfunction, and cellular senescence [108]. Moreover, Jin identified transcription factor Gfi1 as downregulated in OA; its restoration suppresses MAPK activation and mitigates chondrocyte ferroptosis, leading to improved joint structure and function, indicating that MAPK signaling also participates in the regulation of ferroptosis [109]. Collectively, these findings highlight MAPK signaling as one of the most promising and versatile therapeutic nodes in current OA drug and target discovery efforts.

### 4.3 Wnt/β‐Catenin Signaling Pathway

The Wnt/β‐catenin pathway is a developmental signaling pathway that modulates cell differentiation and tissue remodeling. This pathway is pathologically reactivated in OA and contributes to joint destruction in this disease. Abnormal Wnt/β‐catenin signaling leads to a loss of chondrocyte phenotype and matrix homeostasis: nuclear β‐catenin forms complexes with T‐cell factor (TCF)/lymphoid enhancer‐binding factor transcription factors, driving the expression of catabolic enzymes including MMP13 and ADAMTS5, as well as factors that promote hypertrophy such as RUNX2 [6]. In adult articular cartilage and osteochondral units, continuous Wnt/β‐catenin activation accelerates chondrocyte proliferation while pushing cells toward terminal differentiation into a hypertrophic fate, primarily mediated through increased expression of canonical target genes c‐Myc and Cyclin D1, which induce degenerative changes [110]. In humans, single nucleotide polymorphisms in Wnt receptors and coreceptor genes have been suggested by Ward et al. to increase the risk of knee and hip OA. In experimental animal models, both Wnt/β‐catenin overactivation and complete inhibition worsen joint degeneration, supporting the concept that OA progression depends on a strictly defined “permissive window” of Wnt activity [111]. In injury‐induced OA animal models, aberrant activation of Wnt/β‐catenin signaling has also been described in the synovium. Following joint injury, proteoglycan 4 (PRG4)‐expressing synovial fibroblasts secrete the Wnt agonist R‐spondin‐2, promoting pathological crosstalk among synovial fibroblasts, macrophages, and chondrocytes and thereby contributing to OA progression [112]. Various Wnt ligands play distinct roles in OA, forming diverse regulatory networks (Table 1).

**TABLE 1:** Key Wnt‐signaling molecules and their functions in OA.

Wnt	Main findings	OA‐associated functional roles	References
Wnt16	Genetic deletion of Wnt16 aggravates DMM‐induced OA, characterized by reduced PRG4 expression, increased chondrocyte apoptosis, and enhanced canonical Wnt/β‐catenin signaling.	Protective: restrains excessive canonical Wnt activation and preserves superficial zone integrity	[113]
Wnt16	Loss of Wnt16 exacerbates anterior cruciate ligament transection (ACLT)‐induced cartilage degeneration, whereas intra‐articular Wnt16 delivery attenuates OA progression via the PCP/JNK–mTORC1–PTHrP axis, suppressing chondrocyte hypertrophy.	Protective/antihypertrophic	[114]
Wnt7a	Wnt7a expression is inversely correlated with catabolic markers in human OA cartilage; exogenous Wnt7a suppresses IL‐1β‐induced MMP and iNOS expression and alleviates cartilage damage in DMM models.	Protective/anticatabolic	[115]
Wnt5a	OA‐related stimuli induce Wnt5a expression in chondrocytes; recombinant Wnt5a upregulates MMP1/3/13 and suppresses matrix anabolic genes through activation of noncanonical MAPK/JNK pathways.	Procatabolic/prodegenerative	[116]
Wnt3a	In OA‐like chondrocyte models, Wnt3a knockdown reduces β‐catenin and MMP13 expression while restoring COL2A1, indicating involvement of Wnt3a/β‐catenin signaling in cartilage catabolism.	Prodegenerative (canonical Wnt pathway)	[117]
Wnt9a	Transcriptomic and phenotypic analyses identify Wnt9a as an OA‐associated gene; Wnt9a expression is elevated in lesioned compared with preserved human OA cartilage.	Lesion‐associated/disease‐promoting tendency	[118]
Wnt9a/Wnt4	Conditional deletion of Wnt9a or Wnt4 leads to spontaneous age‐related OA‐like joint changes in mice; combined loss results in more severe cartilage and bone abnormalities.	Protective: maintenance of joint homeostasis	[119]
Wnt10a	Epigenetic regulation by KDM6A modulates Wnt10a/Fzd10 transcription; inhibition of KDM6A attenuates DMM‐induced cartilage destruction, synovitis, and osteophyte formation.	Prodegenerative (epigenetic–Wnt axis)	[120]

Abbreviations: FZD10, frizzled class receptor 10; KDM6A, lysine demethylase 6A; PCP, planar cell polarity; PTHrP, parathyroid hormone‐related protein.

Given the disease‐promoting role of Wnt/β‐catenin signaling in OA, recent studies have explored therapeutic strategies targeting this pathway. Experimental evidence shows that genetic ablation of insulin‐like growth factor 1 (IGF‐1) in chondrocytes protects mice from Wnt‐driven joint damage, suggesting that inhibiting Wnt‐induced IGF‐1 production may attenuate OA pathology [121]. In addition, a targeted delivery approach using the inhibitor molecular suppressor of β‐catenin loaded onto human serum albumin‐based nanocarriers and administered intra‐articularly has been reported to enhance β‐catenin degradation and downregulate the downstream target discoidin domain receptor 2, thereby suppressing cartilage destruction and alleviating OA progression and pain‐related symptoms [122]. The first Wnt pathway small‐molecule inhibitor, SM04690 (lorecivivint), inhibits the kinases CDC‐like kinase 2 (CLK2) and dual‐specificity tyrosine phosphorylation‐regulated kinase 1A, indirectly reducing β‐catenin‐dependent transcriptional activity. It promotes mesenchymal stem cell (MSC) chondrogenesis in vitro and enhances cartilage regeneration while limiting cartilage breakdown in animal models [123]. Moreover, other Wnt pathway inhibitors, such as XAV‐939 and C113, have also demonstrated protective effects in murine OA models by reducing cartilage damage and synovial inflammation [124].

However, although chondrocyte hypertrophy, ECM degradation, and OA have all been correlated with aberrant activation of the canonical Wnt/β‐catenin signaling pathway, accumulating evidence suggests that this pathway does not act as a pathogenic “on–off” switch. Studies with drugs and genetic models have shown that both prolonged hyperactivation and complete inhibition of Wnt/β‐catenin can worsen joint pathology, implying that the maintenance of cartilage homeostasis depends on a relatively small range of Wnt signaling activity [125]. This may be reconcilable with disease stage, signal intensity, and cell type‐specific effects. Under normal joint conditions, or during repair of early injury, a degree of Wnt signaling is necessary for progenitor cell function of superficial zone cells, as well as for the expression of lubricants and repair tissue. Chronic inflammation and mechanical stress, however, perturb Wnt/β‐catenin signaling, usually engendering terminal chondrocyte differentiation and catabolic programs [126]. Moreover, proteins that exist outside what is considered the canonical Wnt pathway (e.g., Wnt5a, Wnt11) often induce biological consequences opposite to those stemming from the canonical pathway by acting through β‐catenin‐independent mechanisms, complicating approaches that simply seek to globally inhibit Wnt signaling [127]. Overall, the current understanding supports the notion that Wnt signaling should be fine‐tuned in a time‐ and tissue stage‐dependent manner rather than completely blocked in the context of OA, a perspective that has direct implications and cautions regarding the clinical translatability of Wnt‐directed strategies.

### 4.4 PI3K–AKT–mTOR Signaling

**FIGURE 5:**
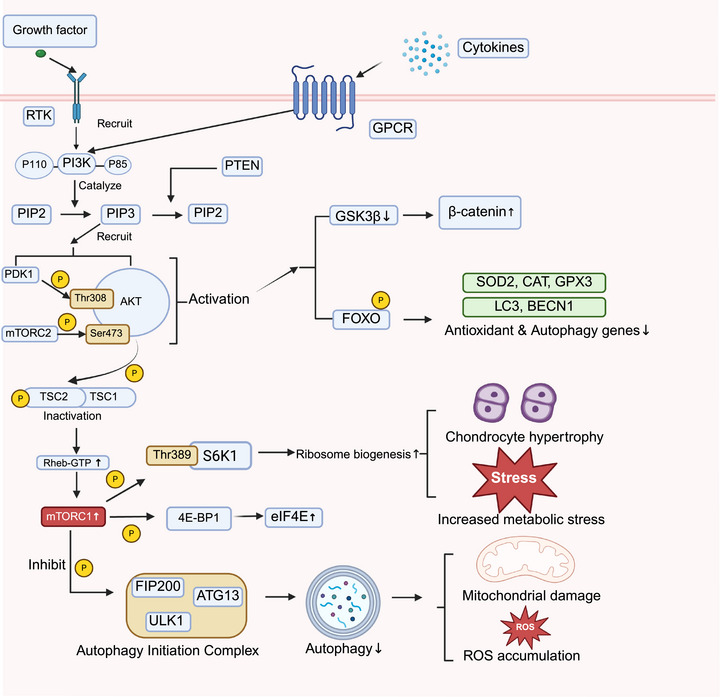
PI3K–AKT–mTOR signaling in chondrocyte dysfunction during osteoarthritis. Activation of the PI3K–AKT–mTOR pathway disrupts redox balance and autophagy, promoting metabolic stress, mitochondrial dysfunction, and cartilage degeneration in osteoarthritis. (Created with BioRender.com.)

The PI3K–AKT–mTOR pathway is a central regulatory axis controlling cellular energy metabolism, redox balance, and autophagy, and is persistently activated in OA, spanning the continuum of inflammation, metabolic dysregulation, and structural remodeling. In OA joints, diverse stimuli activate this pathway, including proinflammatory cytokines (e.g., IL‐1β and TNF‐α), excessive mechanical loading, growth factors such as IGF‐1 and transforming growth factor beta (TGF‐β), as well as oxidative stress and adipokines. Upon activation of upstream receptor tyrosine kinases or G protein‐coupled receptors, PI3K generates phosphatidylinositol (3,4,5)‐trisphosphate (PIP3), leading to membrane recruitment and activation of AKT by 3‐phosphoinositide‐dependent protein kinase 1, and mechanistic target of rapamycin complex 2 (mTORC2). Activated AKT subsequently inhibits tuberous sclerosis complex 1/2, thereby promoting Ras homolog enriched in brain–mTORC1 signaling, which regulates protein synthesis, cellular metabolism, and autophagy (Figure 5) [128, 129].

Recent studies on PI3K–AKT–mTOR signaling in OA have increasingly focused on how fine‐tuning this pathway can restore chondrocyte autophagy and antioxidant capacity. The metabolic modulator 4‐octyl itaconate (4‐OI) suppresses PI3K–AKT–mTOR signaling, enhances autophagic flux, reduces ROS accumulation and ECM degradation, and thereby improves cartilage structure in murine OA models [130]. Similarly, icariin induces chondrocyte autophagy via the PI3K–AKT–mTOR/ULK1 axis and attenuates inflammatory injury, exerting stable chondroprotective effects both in vitro and in vivo [131]. At the level of mitochondrial homeostasis, overexpression of sirtuin 3 (SIRT3) inhibits IL‐1β‐induced PI3K–AKT–mTOR activation, restores autophagy, and alleviates mitochondrial dysfunction and chondrocyte apoptosis, highlighting the “mitochondrial deacetylase–PI3K–mTOR” axis as a key link between energy metabolism and cartilage degeneration [132].

Among clinically translatable metabolic agents, metformin suppresses aberrant PI3K–AKT–mTOR activation in surgically induced OA models, restores autophagy and anabolic–catabolic balance in chondrocytes, and is associated with reduced Mankin scores and improved subchondral bone remodeling, suggesting potential repurposing of antidiabetic drugs for OA treatment [133]. In addition, the natural small molecule perillyl alcohol markedly downregulates p‐PI3K, p‐AKT, and p‐mTOR, increases autophagy markers, and reduces MMP13 and IL‐1β expression in OA chondrocytes, exemplifying a canonical “pathway inhibition–autophagy induction–anticatabolic” pattern [134]. At the nucleic acid level, miR‐103‐3p is upregulated under inflammatory stimulation and activates PI3K–AKT–mTOR signaling, thereby suppressing autophagy and exacerbating chondrocyte apoptosis and ECM degradation. Inhibition of miR‐103‐3p reverses these effects, pointing to the microRNA (miRNA)–PI3K–mTOR axis as a candidate nucleic acid–based therapeutic entry point [135]. Not only pharmacological but also nonpharmacological strategies are being investigated. Combined functional training was found to downregulate pathway activity, improve autophagy and chondrocyte viability, with parallel improvements in joint structure and pain [136]. Platelet‐rich plasma (PRP) with quadriceps strengthening exhibits a similar “autophagy restoration–degeneration attenuation” pattern, further implicating modulation of PI3K–AKT–mTOR signaling as a convergent mechanism for multimodal OA interventions [137].

Importantly, whereas inhibition of the PI3K–AKT–mTOR axis enhances autophagy and reduces oxidative stress, excessive or chronic inhibition of mTOR signaling may impair the anabolic processes required for cartilage matrix synthesis, suggesting that the therapeutic benefits of targeting this pathway depend on balanced modulation rather than continuous pathway inhibition [138].

## 5 Epigenetic Regulatory Mechanisms

Epigenetic regulation in OA provides a key mechanistic framework linking gene‐environment interactions. Without altering the DNA sequence, epigenetic mechanisms—including DNA methylation, histone modifications, and ncRNAs—reprogram transcriptional programs in chondrocytes and synoviocytes, thereby driving inflammatory amplification, ECM degradation, and cellular senescence. Among these mechanisms, histone modifications represent a major epigenetic regulatory layer in eukaryotic cells. As core nuclear proteins, histones package DNA into nucleosomal structures [139]. Their N‐ and C‐terminal tails undergo diverse posttranslational modifications, including acetylation, methylation, phosphorylation, and ubiquitination [140].

### 5.1 DNA Methylation

**FIGURE 6:**
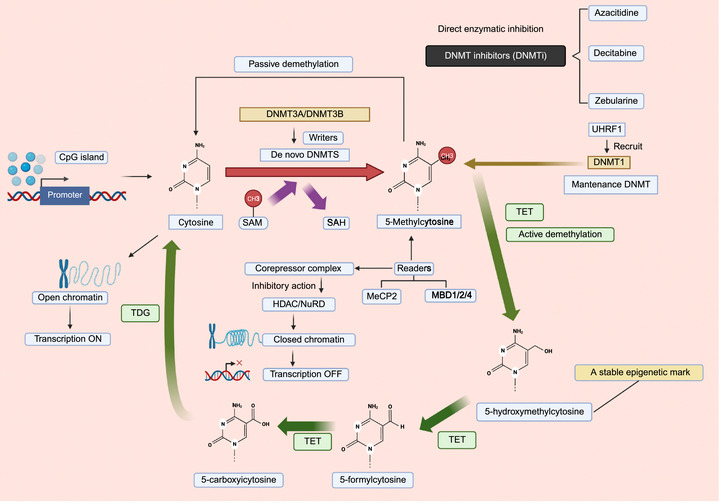
Dynamic regulation of DNA methylation and demethylation. DNA methylation deposited by DNMTs promotes chromatin compaction and transcriptional repression, whereas TET‐mediated demethylation restores chromatin accessibility and gene expression. (Created with BioRender.com.)

DNA methylation is one of the most extensively studied epigenetic modifications and primarily occurs at cytosine–phosphate–guanine (CpG) dinucleotides in DNA. This process is catalyzed by DNA methyltransferases (DNMT1, DNMT3A, and DNMT3B), which add a methyl group to the 5‐position of cytosine to form 5‐methylcytosine (5‐mC). In contrast, members of the ten–eleven translocation (TET) family can oxidize 5‐mC to intermediates such as 5‐hydroxymethylcytosine (5‐hmC), thereby participating in active DNA demethylation processes [141]. In addition, reader proteins such as methyl‐CpG binding domain protein 2 (MBD2) recognize methylated CpG sites and recruit transcriptional repressor or activator complexes [142]. In general, hypermethylation of promoter or enhancer regions is associated with gene silencing, whereas gene body methylation may exert a context‐dependent, potentially transcription‐promoting effect. Therefore, DNA methylation is considered a finely tunable regulatory dial rather than a simple on/off switch (Figure 6).

In OA, the methylomes of articular cartilage and the osteochondral tissues have been shown to differ markedly from those of normal joints. Early DNA methylation array studies demonstrated that OA and healthy cartilage differ at hundreds to thousands of CpG sites, involving pathways related to ECM degradation, inflammation, and cell differentiation, providing initial evidence for OA‐specific methylation signatures [143]. More recently, large‐scale integrative methylome–GWAS studies further revealed that certain OA susceptibility loci influence the expression of key genes in chondrocytes through local alterations in DNA methylation, thereby amplifying or buffering genetic risk in this tissue‐specific context. High‐resolution profiling of articular cartilage methylomes has identified multiple OA GWAS loci overlapping with disease‐associated differentially methylated regions (DMRs), helping to explain the functional consequences of genetic signals [144, 145].

From a mechanistic perspective, DNA methylation can be either protective or pathogenic. On the one hand, cartilage‐specific deletion of DNMT3B leads to reduced matrix synthesis, disordered energy metabolism, and spontaneous cartilage degeneration, whereas appropriate DNMT3B overexpression can alleviate cartilage injury induced by mechanical stress and inflammatory stimulation in vivo and in vitro, suggesting that maintaining physiological methylation levels is part of cartilage homeostasis [145]. On the other hand, single‐locus studies have shown that a specific site within the ADAMTS4 promoter is demethylated in OA cartilage, paralleling a marked (hundreds‐fold) increase in its mRNA expression, indicating that local demethylation–overactivation of catabolic enzymes is a key node driving ECM imbalance [146]. Similarly, the promoter/enhancer methylation status of OA‐related genes such as GDF5 is closely associated with their expression levels and cartilage‐protective effects [147, 148].

The DNA demethylation pathway is also involved in OA progression; TET1‐mediated 5‐hmC deposition is disproportionately enhanced in OA cartilage and enriched at the regulatory regions of numerous metabolism‐ and inflammation‐related catabolic genes. Pharmacological intervention or genetic inhibition of TET1 in murine models reduces aberrant activation of these genes and delays cartilage degeneration, suggesting that an abnormally enhanced demethylation program may be one of the forces driving chondrocytes toward a degradative phenotype [149]. More recent studies have focused on methylation reader proteins: MBD2 is upregulated in OA cartilage, and inhibition of MBD2 can reprogram local methylation and transcriptional programs and attenuate cartilage destruction, suggesting that the coordinated “reader–writer–eraser” machinery might be a viable intervention target [150].

Notably, dysregulated methylation is not confined to cartilage. In an MIA‐induced knee OA model, DNMT3A, TET1, and TET3 are coordinately upregulated in synovial tissue, with a concomitant increase in overall DNA methylation levels that positively correlates with synovitis severity and pain sensitivity, suggesting that, in the setting of prolonged epigenetic reprogramming, synovial inflammation may exhibit more persistent responsiveness [151].

More interestingly, OA‐related dysregulation of DNA methylation is not confined to local joint tissues, as changes are also observed in distant systemic and peripheral compartments. Several studies utilizing 850K genome‐wide DNA methylation arrays in combination with machine‐learning methods have demonstrated that predictions of knee OA onset, imaging progression, or pain trajectories can be derived from models trained on a small number of CpG sites [152]. These findings suggest that OA susceptibility and progression are not determined solely by the joint microenvironment but also reflect epigenetically time‐integrated indicators of systemic immune and metabolic states shaped by long‐term lifestyle exposures. In this sense, blood DNA methylation is not merely a passive biomarker but rather represents a dynamic readout of cross‐tissue epigenetic coordination linking global and local signals.

Overall, the data suggest that OA methylation research is evolving from a descriptive genome‐wide profiling phase toward a more mechanistically driven, stratified phase. Distinct OA methylation subtypes—cartilage‐, synovium‐, or bone‐driven—are characterized by stable CpG/DMRs patterns, each associated with specific cellular compositions and metabolic and inflammatory environments.

### 5.2 Histone Methylation

Among various histone modifications, methylation has emerged as the major research focus in recent years. Unlike DNA mutations, histone methylation can be precisely and reversibly regulated by methyltransferases and demethylases. The dynamic balance of these enzymes is essential for maintaining the homeostasis of bone‐related cells, inflammatory signaling, and ECM metabolism [153].

**FIGURE 7:**
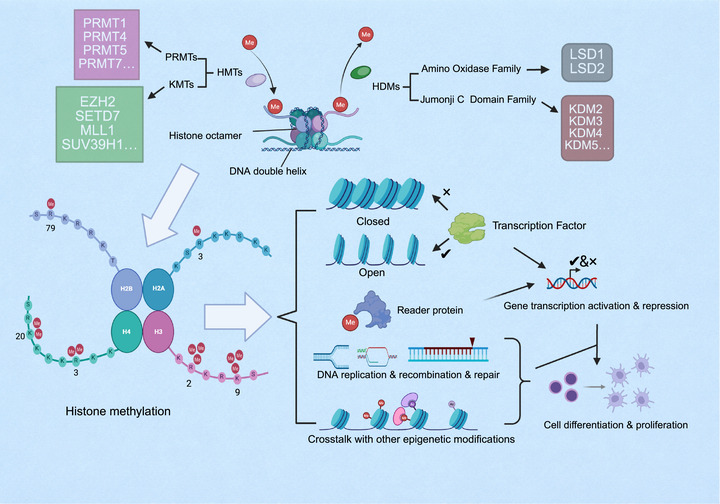
Histone methylation in epigenetic regulation of gene expression. Histone methyltransferases and demethylases dynamically regulate chromatin structure and transcriptional activity, influencing DNA repair, recombination, and cellular differentiation. (Created with BioRender.com.)

The histone methylation system consists of both methyltransferases and demethylases, which precisely regulate chromatin structure and gene expression through dynamic and reversible chemical modifications [154]. Histone methyltransferases are generally classified into two major categories based on substrate specificity: protein arginine methyltransferases (PRMTs) and lysine methyltransferases (KMTs). The PRMT family primarily catalyzes the methylation of histone arginine residues (e.g., H3R2, H3R8, and H4R3), altering nucleosome surface charge and thereby affecting chromatin conformation. This is of great importance for gene transcriptional regulation, chromatin stability and transmission, and cellular signal transduction. The KMT family catalyzes methylation of histone lysine residues (e.g., H3K4, H3K27, H3K36, and H4K20). Different lysine methylation marks exert distinct biological functions (e.g., H3K4me3 is associated with transcriptionally active regions, whereas H3K27me3 mediates gene silencing), thereby yielding a complex regulatory network (Figure 7) [155, 156, 157, 158].

The histone demethylase system serves to preserve the dynamic balance of epigenetic modifications by removing methyl marks from histones, and its functional classification is analogous to that of histone methyltransferases. Histone demethylases are chiefly divided into two major families based on their catalytic mechanisms and structures. The lysine‐specific demethylase (LSD) family, represented by LSD1, comprises flavin adenine dinucleotide‐dependent enzymes that primarily demethylate mono‐ and di‐methylated H3K4 and H3K9 residues through an amine oxidation reaction [159]. Another family is the Jumonji C domain‐containing demethylases (JmjC family). Members of this family contain a conserved JmjC domain. These enzymes utilize Fe^2^^+^ and α‐ketoglutarate as cofactors and can remove mono‐, di‐, and tri‐methyl groups from a variety of histone lysine residues such as H3K4, H3K9, H3K27, and H3K36 [160]. Both classes of demethylases are important in regulating chromatin conformation, gene transcriptional activity, and cell fate determination, and dysregulation of these enzymes is associated with several diseases.

Therefore, we examine histone methylation from the perspective of distinct OA‐relevant cell types and pathological processes, integrating evidence from chondrocyte phenotypic alterations and degeneration, synovial inflammation, inflammation‐associated ECM degradation, and subchondral bone remodeling to delineate its mechanistic roles in OA.

#### 5.2.1 Chondrocyte Differentiation

PRMT4 regulates the expression of cell cycle genes such as Cyclin D1 by methylating the transcription factor SRY‐box transcription factor 9 (SOX9), thereby promoting chondrocyte proliferation and differentiation. Loss of PRMT4 function inhibits chondrocyte differentiation and hastens endochondral ossification [161]. The activity of PRMT5 depends on trace elements. Under selenium‐replete conditions, PRMT5 stabilizes SOX9 protein, leading to increased synthesis and decreased degradation of the cartilage matrix. Selenium deficiency impairs cartilage regulation through this mechanism [162]. The H3K79 methyltransferase disruptor of telomeric silencing 1‐like (DOT1L) is essential for chondrocyte differentiation, matrix production, and homeostasis in the growth plate; its knockout results in inhibited differentiation and matrix production, as well as alterations in bone remodeling and density, together with downregulation of cartilage marker genes such as aggrecan (ACAN) and COL2A1 [163]. Large‐scale GWAS and functional studies have confirmed that DOT1L gene polymorphisms are significantly associated with cartilage thickness and risk of hip OA [164]. Molecularly, DOT1L‐catalyzed H3K79 methylation is regulated by hypoxia‐inducible factor 1‐alpha (HIF1A): upregulation of HIF1A not only promotes DOT1L expression but also increases H3K79 methylation, together suppressing aberrant activation of the Wnt/β‐catenin pathway and maintaining chondrocyte phenotype and matrix metabolic balance [165]. Enhancer of zeste homolog 2 (EZH2) maintains H3K27me3 levels to repress the expression of osteogenic genes, ensuring the stability of chondrocyte differentiation. Although deletion of EZH2 significantly activates osteogenic gene expression and promotes osteoblast‐like differentiation, its impact on articular cartilage development and OA‐like pathology appears limited [166]. In addition, the lncRNA CIR recruits EZH2 to enhance H3K27me3 modification at the ATOH8 promoter, thereby suppressing its expression and hindering chondrogenic differentiation of human umbilical cord MSCs [167]. Of significance, SET domain bifurcated histone lysine methyltransferase 1 (SETDB1), an H3K9 methyltransferase, is highly expressed in articular cartilage and is essential for maintaining the differentiated chondrocyte phenotype: mesenchyme‐specific knockout does not affect embryonic cartilage development, but postnatal deletion leads to premature chondrocyte hypertrophy, increased terminal differentiation and apoptosis, upregulation of MMP13, and matrix degeneration, ultimately exacerbating cartilage damage [168]. In addition, nuclear receptor binding SET domain protein 1 (NSD1) expression is decreased in the cartilage of older individuals and in the cartilage of individuals with age‐related OA. The absence of this gene produces spontaneous cartilage damage and resembles OA phenotypes due to reduced anabolic activity, increased catabolism, and impaired differentiation of chondrocytes. Mechanistically, NSD1 maintains cartilage homeostasis by mediating H3K36 methylation to regulate the expression of the transcription factor odd‐skipped‐related transcription factor 2 (OSR2), and exogenous supplementation of OSR2 can partially rescue the abnormal phenotype caused by NSD1 deficiency [169].

LSD1 (also known as KDM1A) is highly expressed in OA chondrocytes and suppresses the transcriptional activity of collagen type IX alpha 1 chain (COL9A1) through cooperation with the transcription factor SOX9, thereby impairing cartilage matrix synthesis [170]. This enzyme also maintains chondrocyte function by regulating key factors such as nuclear factor of activated T cells 1 (NFAT1); downregulation or loss of LSD1 activity leads to dysregulation of matrix metabolism and exacerbates OA degeneration [171]. Additionally, lysine demethylase 3A (KDM3A) promotes chondrocyte differentiation and phenotype maintenance by upregulating core components of the Wnt/β‐catenin pathway and downstream genes such as B‐cell lymphoma 6 (BCL6) and Jun proto‐oncogene (JUN) in OA models [172]. Knockdown of lysine demethylase 4B (KDM4B) significantly reduces the differentiation potential of chondrocytes and diminishes SMAD3 binding to the SOX9 promoter, thereby blocking TGF‐β/SMAD pathway‐mediated activation of chondrogenic genes [173]. Lysine demethylase 5C (KDM5C) expression is regulated by the lysyl oxidase like 1 antisense RNA 1 (LOXL1–AS1)/miR‐423‐5p axis, and increased KDM5C activity enhances the proliferative and differentiation capacity of chondrocytes [174]. Lysine demethylase 6A (KDM6A) activates chondrogenic genes such as SOX9, COL2A1, and ACAN by removing the repressive H3K27me3 mark, thereby maintaining the chondrocyte phenotype and matrix synthesis; its loss aggravates OA cartilage degeneration, suppresses the expression of chondrocyte markers, enhances H3K27me3‐mediated transcriptional repression, and activates Wnt10a signaling pathways, which disrupts joint homeostasis [120, 175]. KDM6A expression is downregulated through a CircNFIX (circular RNA [circRNA] NFIX)‐dependent regulatory mechanism, leading to impaired chondrogenic differentiation and reduced ECM synthesis, thereby accelerating disease progression [176]. In mouse models with chondrocyte‐specific knockout of KDM6B, both embryonic and adult mice exhibit reduced cartilage development and diminished expression of anabolic genes, resulting in abnormal cartilage structure and impaired function. Further studies in OA models have confirmed that loss of KDM6B leads to more severe articular cartilage degeneration and significantly higher OA scores [177]. Other studies, however, demonstrate that KDM6B expression is upregulated in response to inflammatory stimuli in OA, and by removing repressive marks from bivalent genes (marked by both H3K27me3 and H3K4me3), it relieves transcriptional repression of developmental genes and induces chondrocyte transdifferentiation toward a growth plate‐like hypertrophic phenotype [178].

**FIGURE 8:**
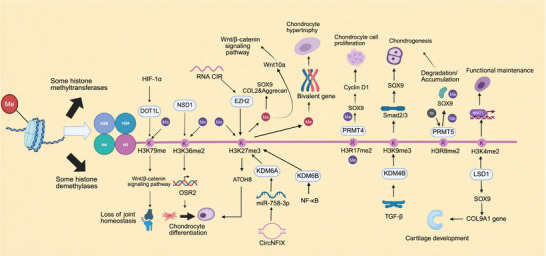
Histone methylation regulates chondrocyte differentiation and cartilage homeostasis. Histone methyltransferases and demethylases coordinate transcriptional programs that control chondrocyte proliferation, hypertrophy, and cartilage development through interactions with key signaling pathways. (Created with BioRender.com.)

Collectively, histone methylation emerges as a key epigenetic determinant of chondrocyte differentiation by stabilizing cartilage‐specific transcriptional programs and restraining aberrant hypertrophic and catabolic activation. Disruption of this finely balanced methylation landscape predisposes chondrocytes to phenotypic instability, matrix degradation, and progressive cartilage degeneration during OA (Figure 8).

#### 5.2.2 Inflammation and Matrix Degradation

PRMT1 promotes IL‐1β‐induced ECM degradation and chondrocyte apoptosis by regulating the AKT/forkhead box O1 (FOXO1) signaling pathway [179]. Inflammatory stimuli can upregulate PRMT1 activity via the lncRNA PILA (proinflammatory lncRNA associated with OA), thereby enhancing NF‐κB signaling and matrix degradation in chondrocytes and accelerating OA progression [180]. PRMT1 is highly expressed in OA cartilage and in chondrocytes under inflammatory stimulation, where it promotes IL‐1β‐induced release of inflammatory cytokines and the expression of matrix‐degrading enzymes. It also exacerbates inflammation and ECM destruction by activating the Hedgehog/Gli‐1 pathway. Knockdown of PRMT1 significantly suppresses inflammation and matrix degradation, conferring a protective effect on cartilage [181]. Similarly, under stimulation by inflammatory cytokines such as IL‐1β, PRMT5 is upregulated in OA chondrocytes, where it promotes matrix degradation by activating the NF‐κB and MAPK signaling pathways. Inhibition of PRMT5 effectively alleviates inflammation‐induced cartilage injury [182]. EZH2 is upregulated in OA cartilage and within the inflammatory microenvironment, and it significantly amplifies IL‐1β‐induced chondrocyte inflammatory responses, thereby aggravating cartilage damage [183]. The H3K4 methyltransferase SET‐1A is also upregulated in OA chondrocytes and is recruited to the promoters of iNOS and COX‐2, promoting H3K4 di‐ and tri‐methylation and enhancing transcription of inflammatory genes [184]. In addition, circSCAPER (circRNA SCAPER) is significantly upregulated in OA cartilage and under inflammatory conditions, where it acts as a sponge for miR‐140‐3p, thereby relieving the repression of EZH2, activating EZH2 expression, and further enhancing PI3K–AKT signaling. This promotes matrix degradation, impaired proliferation, and increased apoptosis of chondrocytes in response to inflammatory stimuli [185]. Interestingly, some studies have shown that EZH2 can upregulate TNF superfamily member 13B (TNFSF13B) expression, activate the PI3K–AKT signaling pathway, promote chondrocyte repair, and inhibit hypertrophic differentiation, thus slowing OA progression. Chondrocyte‐specific deletion of EZH2 leads to aggravated joint damage, reduced repair capacity, and increased hypertrophic phenotype, while exogenous supplementation with TNFSF13B can partially reverse these phenotypes [186]. These findings underscore epigenetic factors may exert bidirectional and complex regulatory effects depending on the experimental model or stage of disease.

LSD1‐mediated H3K9 demethylation is a key epigenetic mechanism in the inflammatory response of OA chondrocytes. IL‐1β stimulation promotes the recruitment of LSD1 to the microsomal PGE synthase‐1 (mPGES‐1) promoter region, leading to its transcriptional activation and increased PGE2 synthesis, thereby exacerbating chondrocyte inflammation and cartilage damage [187]. KDM6B expression is markedly upregulated in OA chondrocytes under inflammatory stimulation. The activity of KDM6B is regulated by S‐phase kinase‐associated protein 2 (SKP2)‐mediated degradation of Krüppel‐like factor 11 (KLF11), an upstream negative regulator. KDM6B promotes the expression of proinflammatory cytokines and matrix‐degrading enzymes through H3K27me3 demethylation, and its inhibition alleviates inflammatory injury in OA models [188]. Under abnormal mechanical stress, KDM6B is also significantly upregulated and removes H3K27me3 marks from the promoters of target genes, promoting the expression of nuclear receptor subfamily 4 group A member 1 (NR4A1) and thereby driving ECM degradation and activation of the inflammatory response in chondrocytes [189].

#### 5.2.3 Regulation of Synoviocyte Changes

Previous studies have shown that the high expression of the inflammatory cytokine IL‐6 in OA synoviocytes primarily depends on epigenetic modifications such as DNA hypomethylation and increased histone acetylation in the promoter region, while the levels of histone methylation marks such as H3K9me2, H3K27me3, and H3K4me3 do not differ significantly between OA and normal synoviocytes. These findings suggest that histone methylation may not be a key factor in the regulation of synovial inflammation and the abnormal activation of inflammatory mediators such as IL‐6, and that the underlying mechanisms and the specific functions of related enzymes require further investigation [190, 191]. Some studies have also demonstrated that EZH2 is expressed in OA synoviocytes and can be upregulated in response to inflammatory stimuli. EZH2 can suppress the expression of secreted frizzled‐related protein 1 (SFRP1), an inhibitor of the Wnt pathway, via H3K27me3 modification, thereby enhancing the sensitivity of synoviocytes to Wnt signaling and promoting synoviocyte activation and joint destruction under inflammatory conditions [192]. In fact, the site‐specific functions of synovial fibroblasts are closely associated with modifications such as H3K27me3, regulated by EZH2 and KDM6B. These enzymes modulate the epigenetic status of positional genes such as homeobox (HOX) genes, endowing synoviocytes with differential inflammatory and matrix degradation responses, and contribute to the site susceptibility of OA synovitis [193]. Extracellular vesicles derived from synovial MSCs can suppress inflammation and OA progression by downregulating the histone demethylase lysine demethylase 2A (KDM2A) through miR‐31 and regulating E2F transcription factor 1 (E2F1)/pituitary tumor‐transforming gene 1‐related signaling [194].

#### 5.2.4 Aberrant Regulation of Subchondral Bone Remodeling

Abnormal subchondral bone remodeling—such as sclerosis or excessive resorption—is a key event in the progression of OA, and its pathological changes mutually promote the degeneration of the overlying cartilage [74]. In subchondral bone osteocytes, H19 enhances EZH2‐mediated H3K27me3, modulates the osteocytic response to mechanical stimulation, alters PI3K–AKT signaling and the expression of osteogenesis‐related genes, and thereby promotes aberrant subchondral bone remodeling during OA progression [195]. LSD1 regulates osteoclast differentiation and bone resorption, significantly impacting bone remodeling. In the inflammatory microenvironment and under hypoxic conditions, LSD1 expression is upregulated, which promotes HIF‐1α stabilization and activation of glycolytic metabolism, thereby accelerating osteoclast differentiation and functional maturation. LSD1 can also enhance RANKL signaling pathway activity and upregulate transcription factors such as E2F1, coordinately regulating the expression of genes involved in bone resorption [196]. KDM4B is suppressed, leading to reduced expression of the downstream osteogenic transcription factor distal‐less HOX 5 (DLX5). This, in turn, impairs the differentiation of bone marrow MSCs into osteoblasts and thereby regulates subchondral bone formation and remodeling in OA [197].

Taken together, these data support a model in which histone methylation represents a cell type‐specific layer of epigenetic regulation in OA, orchestrating the postnatal spectrum of chondrocyte differentiation, inflammatory activation, ECM turnover, and subchondral bone remodeling. Working in concert rather than in isolation, various histone methyltransferases and demethylases converge on shared transcriptional programs, and remodeling of their balance precipitates degenerative reprogramming of cartilage and disruption of joint homeostasis (Table 2).

**TABLE 2:** Key histone methyltransferases and demethylases involved in OA pathogenesis and their molecular functions.

Enzyme	Target site	Main cell type(s)	OA pathological process	Key pathway/axis	Main molecular function	References
PRMT1	H3R17me2	Chondrocyte	Inflammation, ECM degradation	AKT/FOXO1, NF‐κB, Hedgehog	Promotes proinflammatory cytokine and MMP expression	[179, 180, 181]
PRMT4	H3R17me2, Sox9	Chondrocyte	Differentiation	Cyclin D1, SOX9	Promotes chondrocyte proliferation and differentiation	[161]
PRMT5	H3R8me2, H4R3me2	Chondrocyte	Differentiation, inflammation, ECM metabolism	SOX9, NF‐κB, MAPK	Stabilizes SOX9, inhibits ECM degradation	[162, 182]
DOT1L	H3K79me2/3	Chondrocyte	Differentiation, ECM synthesis, inflammation	Wnt/β‐catenin	Maintains cartilage phenotype, suppresses catabolism	[163, 164, 165]
EZH2	H3K27me3	Chondrocyte, synoviocyte, osteoblast	Differentiation, inflammation, ECM degradation, synovitis	PI3K–AKT, miR‐138/SDC1, TNFSF13B	Represses anabolic gene expression, promotes catabolism and inflammation (dual role)	[166, 167, 183, 186]
NSD1	H3K36me2	Chondrocyte	Differentiation, ECM synthesis	OSR2, SOX9	Promotes ECM anabolism, maintains chondrocyte phenotype	[169]
SETDB1	H3K9me3	Chondrocyte	Differentiation	—	Maintains differentiated chondrocyte phenotype, prevents hypertrophy	[168]
LSD1 (KDM1A)	H3K4me1/2, H3K9me1/2	Chondrocyte, osteoclast	Differentiation, inflammation, ECM degradation	SOX9, NFAT1	Suppresses anabolic genes, promotes inflammation	[170, 171, 196]
KDM3A	H3K9me2	Chondrocyte	Differentiation, ECM metabolism	BCL6/JUN, Wnt/β‐catenin	Maintains chondrocyte function and phenotype	[172]
KDM4B	H3K9me3, H3K36me3	Chondrocyte, MSC	Differentiation, osteogenesis, bone remodeling	SMAD3, DLX5	Promotes chondrogenic and osteogenic differentiation	[173, 197]
KDM5C	H3K4me3	Chondrocyte	Differentiation	lncRNA LOXL1–AS1/miR‐423‐5p	Promotes chondrocyte proliferation and differentiation	[174]
KDM6A	H3K27me3	Chondrocyte	Differentiation, metabolism	Igf2/PRC2	Activates anabolic metabolism, maintains chondrocyte phenotype	[120, 175, 176]
KDM6B	H3K27me3	Chondrocyte, synoviocyte	Inflammation, ECM degradation, hypertrophy	NOTCH1, Wnt10a, SKP2/KLF11, NR4A1	Activates catabolic/inflammatory genes	[177, 178, 188, 189]

#### 5.2.5 Emerging Roles of Histone Methylation in OA Pathogenesis: Beyond Classical Pathways

**FIGURE 9:**
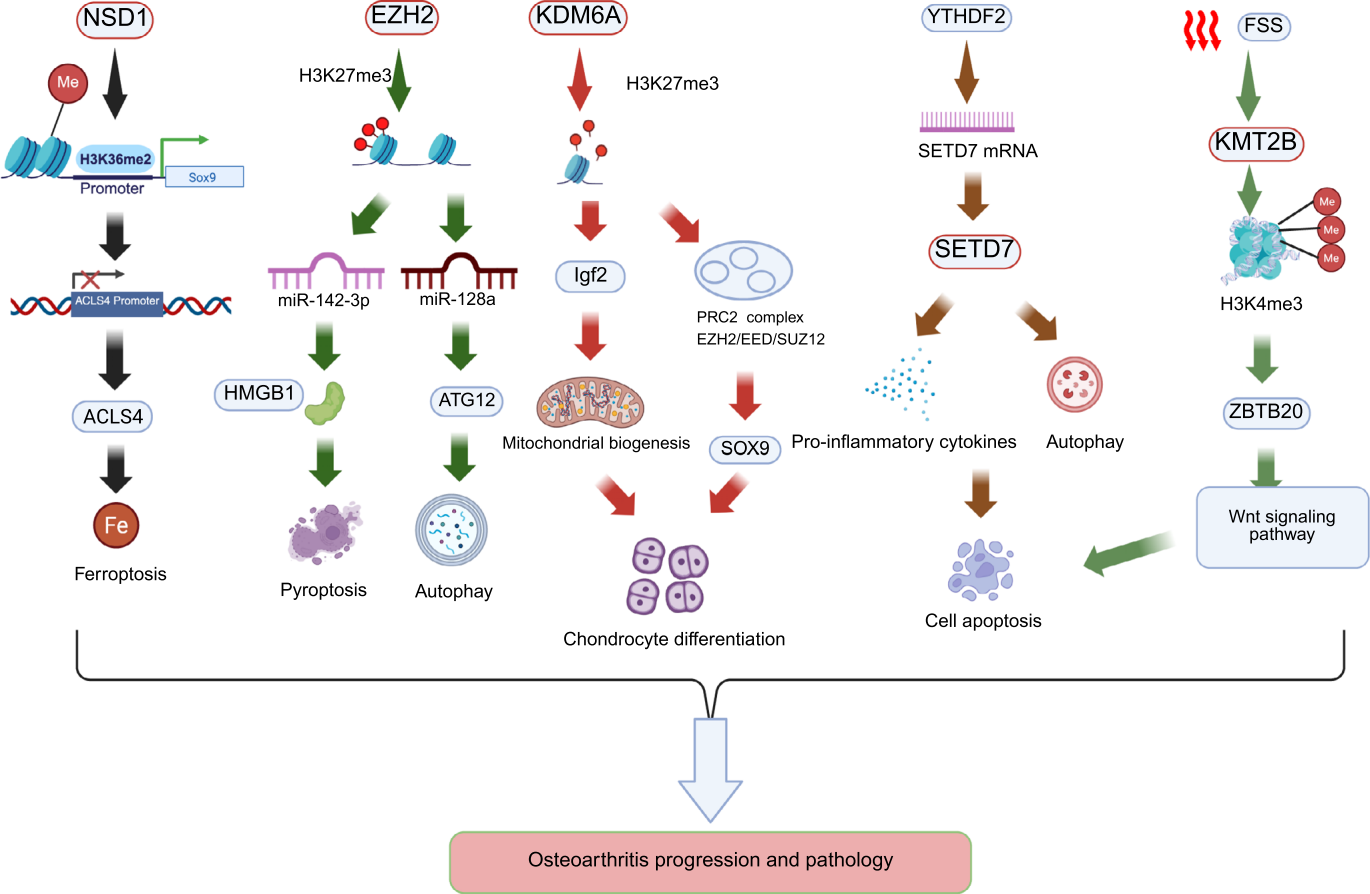
Histone methylation regulators in osteoarthritis pathogenesis. Epigenetic regulators of histone methylation coordinate multiple signaling pathways and cellular processes, including autophagy, inflammatory responses, and chondrocyte differentiation, thereby contributing to osteoarthritis progression. (Created with BioRender.com.)

Recent investigations into OA pathophysiology have revealed a series of mechanisms underlying cell injury and functional regulation associated with this disease. Ferroptosis, pyroptosis, mitochondrial energy metabolism reprogramming, mechanical stress signaling, and dysregulated angiogenesis have been not infrequently implicated in OA. Particularly, histone methyltransferases and demethylases are not only key modulators of these mechanisms but also regulate chondrocyte survival, metabolic homeostasis, and the matrix environment through several axes and signaling pathways (Figure 9).

Ferroptosis is an iron‐dependent mode of programmed cell death mediated by lipid peroxidation. Recent research has revealed its involvement in degenerative diseases such as OA. Ferroptosis is characterized by oxidative damage to membrane lipids and is related to several degenerative diseases and cancers. Xiong et al. found that the histone methyltransferase NSD1 is associated with H3K36me2 methylation at the SOX9 promoter. Increased SOX9 expression subsequently downregulates acyl‐CoA synthetase long‐chain family member 4 (ACSL4), a ferroptosis driver, effectively inhibiting ferroptosis in chondrocytes. The authors also showed that with reduced NSD1 expression, SOX9 levels decreased and ACSL4 increased, resulting in enhanced ferroptosis and cartilage damage that exhibited an OA phenotype. These data not only confirm the involvement of ferroptosis in OA but also highlight the potential for the NSD1–H3K36me2–SOX9–ACSL4 pathway to serve as a therapeutic target [198, 199].

In OA chondrocytes, upregulation of EZH2 suppresses the expression of miR‐142‐3p through H3K27me3‐mediated epigenetic modification, thereby relieving the transcriptional inhibition of high mobility group box 1 (HMGB1) and resulting in a significant increase in HMGB1 expression. Notably, HMGB1 functions not only as a mediator of inflammation but also as a key molecule activating ER stress and pyroptosis pathways. Elevated HMGB1 levels aggravate ER stress and induce pyroptosis, further promoting inflammatory injury in chondrocytes. Interventions targeting EZH2 or HMGB1 activity, or upregulating miR‐142‐3p, can effectively alleviate ER stress and pyroptosis, mitigate OA cartilage degeneration, and highlight the therapeutic potential of targeting the EZH2–HMGB1–pyroptosis axis in OA prevention and treatment [200].

EZH2 can also maintain chondrocyte autophagy and homeostasis by catalyzing H3K27 methylation to effectively repress the transcription of miR‐128a. Autophagy is a highly conserved process of cellular self‐degradation and recycling that maintains cellular homeostasis and stress adaptation by engulfing and degrading damaged organelles or proteins [201]. In OA or under inflammatory stimulation, EZH2 expression and H3K27me2 levels are downregulated, releasing epigenetic suppression of miR‐128a, resulting in miR‐128a upregulation, which subsequently targets autophagy‐related 12 to inhibit autophagy, promoting chondrocyte apoptosis and matrix degradation, and exacerbate OA progression. This mechanism further expands the understanding of the molecular basis of autophagy impairment in OA [202, 203].

In addition, the epigenetic mechanisms underlying mechanical stress have also been gradually elucidated. Abnormally high fluid shear stress (FSS) can upregulate KMT2B‐mediated H3K4me3, activate zinc finger and BTB domain containing 20 (ZBTB20) expression and the Wnt signaling pathway, induce chondrocyte apoptosis, inflammation, and matrix degradation, ultimately aggravating OA‐like pathology. Pharmacological inhibition of H3K4me3 or Wnt signaling significantly alleviates FSS‐induced cartilage injury, demonstrating a novel mechanism by which mechanical signals contribute to OA pathogenesis through epigenetic modifications [204].

Recent genetic studies in the Han Chinese population have shown that polymorphisms such as rs3815308 in the DOT1L gene are closely associated with knee OA susceptibility. Specifically, this coding region missense variant confers a higher risk of OA in carriers of the risk allele. Such genetic variants may alter DOT1L transcription or functional activity, resulting in altered H3K79 methylation and thereby deregulating the activation of downstream critical pathways such as Wnt/β‐catenin, as well as disrupting chondrocyte differentiation homeostasis. Evidence of their functional impact suggests that abnormal DOT1L activity or expression adversely affects cartilage matrix production and maintenance, leading to accelerated OA phenotypes. Thus, the pathogenic effects of DOT1L polymorphisms extend beyond the genetic level and act through epigenetic mechanisms across molecular, cellular, and tissue levels, linking genetic susceptibility to OA phenotypes [205, 206].

KDM6A, an H3K27 demethylase, is upregulated in OA cartilage. It not only regulates differentiation factors but also extensively modulates mitochondrial function, allowing metabolic homeostasis, and therefore acts in a way distinct from other metabolic regulators considered previously. Mechanistically, KDM6A deficiency leads to a decrease in H3K27me3, upregulating IGF2 and thereby promoting mitochondrial biogenesis and energy metabolism, along with repression of the expression of core components of the polycomb repressive complex 2 (PRC2) complex. This, in turn, stimulates the anabolic activity of chondrocytes and helps maintain cartilage homeostasis. These findings link KDM6A to mitochondrial function, metabolic dysregulation, and OA, underscoring the critical role of epigenetic regulation in maintaining metabolic homeostasis [207].

Studies on SET domain containing lysine methyltransferase 7 (SETD7) have further expanded the functional repertoire of epigenetic enzymes in OA. SETD7 activates the JAK–STAT signaling pathway, markedly enhancing the ability of chondrocytes to regulate angiogenesis and oxygen tension, promoting neovascularization and endothelial cell migration into cartilage, and increasing local oxygen tension. This dual regulation of angiogenesis and oxygen metabolism not only promotes chondrocyte apoptosis and matrix breakdown but also provides a new pathogenic mechanism for OA progression and identifies the vascular–oxygen metabolism–epigenetic axis as a potential new therapeutic target for OA [208, 209].

SETD7 also functionally interacts with the m6A reader protein YTH N6‐methyladenosine RNA binding protein 2 (YTHDF2). YTHDF2 is a key m6A‐binding protein that regulates gene expression homeostasis by binding to and degrading m6A‐modified mRNAs, participating in a variety of physiological and pathological processes [210, 211]. SETD7 promotes chondrocyte apoptosis and suppresses autophagy under inflammatory conditions, thereby aggravating cartilage injury and degeneration. Specifically, upregulation of SETD7 not only increases the expression of oxidative stress and inflammatory markers but also decreases the levels of autophagy‐related proteins. Knockdown of SETD7 reverse these pathological changes and significantly alleviate chondrocyte damage. Further research has demonstrated that YTHDF2 maintains high levels of SETD7 expression by stabilizing SETD7 mRNA, and together, they coordinately regulate the balance of apoptosis and autophagy in OA chondrocytes. This discovery enriches the epigenetic regulatory network in OA and suggests that targeting SETD7 and its upstream m6A modification‐related proteins may provide new molecular targets and intervention strategies for OA therapy [212].

#### 5.2.6 Summary and Discussion

From a pathway‐level standpoint, while various histone methyltransferases and demethylases influence distinct target genes and yield diverse phenotypic consequences, their OA‐modifying effects often converge on a small number of core, reiterated signaling networks, forming what can be termed a paradigm of diverse epigenetic entry points but similar signaling endpoints. Existing reviews and systematic studies nominate (a) NF‐κB and (b) MAPK as key hubs involved in inflammatory amplification and catabolic transcriptional outputs; the phenotypic effects of multiple epigenetic enzymes in OA are ultimately executed either by amplifying or attenuating these inflammatory axes. Examples include that, in human chondrocytes, LSD1 has been shown to participate in the transcriptional activation of mPGES‐1 in an IL‐1β‐dependent manner, highlighting that histone demethylation events can be positioned in line with the effector output of canonical inflammatory pathways. In addition, pharmacological inhibition of EZH2 in animal models can inhibit cartilage degeneration and improve functional outcomes, indirectly supporting a stable association between PRC2–H3K27me3‐dependent regulation and inflammatory or catabolic transcriptional programs [183, 187]. With respect to phenotypic maintenance and aberrant differentiation, the majority of studies similarly indicate that the downstream common explanatory framework of distinct epigenetic enzymes often converges on the rebalancing of key fate‐determining pathways, most notably Wnt/β‐catenin and TGF‐β/SMAD signaling. Evidence centered on DOT1L, for example, suggests that its protective role in maintaining cartilage homeostasis is closely associated with restraining excessive activation of Wnt signaling, a pathway whose central involvement in OA cartilage degeneration and osteophyte formation has been repeatedly emphasized across multiple reviews. Concurrently, the TGF‐β/SMAD pathway, as a principal signaling axis intimately linked to cartilage homeostasis, repair, and pathological outcomes, is frequently invoked to explain the directionality‐dependent effects of different epigenetic perturbations observed across disease stages and experimental models [213, 214]. Additionally, a growing body of evidence proposes that information on cell survival, metabolic regulation, and stress adaptation in OA can be unified under one conceptual framework, and that PI3K–AKT–mTOR‐centered survival and metabolic pathways and mechanotransduction‐modulated signaling networks represent a convergence interface through which epigenetic enzymes regulate the delicate balance between autophagy and apoptosis, metabolic reprogramming, and mechanical responsiveness. Taken together, this is conceptualized as a third (convergence) axis for facilitating cross‐enzyme synthesis of epigenetic regulation in OA [6]. By taking this pathway‐oriented perspective, dispersed lines of evidence can be integrated into three high‐frequency convergent modules: inflammation (NF‐κB/MAPK), phenotypic regulation (Wnt and TGF‐β/SMAD), and survival, metabolism, and stress adaptation (PI3K–AKT–mTOR and related pathways), without reiterating enzyme‐specific mechanistic details. This discussion facilitates cross‐enzyme delineation of shared signaling contexts among distinct epigenetic enzymes while establishing the conceptual basis for combinatorial or pathway‐level therapeutic interventions.

Despite substantial progress in elucidating the roles of histone methyltransferases and demethylases in OA, it is increasingly evident that findings across studies are not always concordant, particularly with regard to several key epigenetic regulators. These discrepancies do not necessarily reflect experimental inconsistency but rather highlight the pronounced context dependency of epigenetic regulation in OA.

EZH2 has most frequently been reported as a prodegenerative factor in OA. Its expression is upregulated in OA cartilage, and pharmacological or genetic inhibition of EZH2 attenuates cartilage damage [183, 215]. However, conflicting findings have also been described. In specific experimental models, EZH2 deficiency aggravates OA, an effect associated with reduced repair capacity linked to TNFSF13B, suggesting that the impact of EZH2 is modulated by disease stage and microenvironmental context [186]. Similar discrepancies have likewise been reported for the H3K27 demethylase KDM6B (also known as JMJD3). KDM6B exhibits a pronounced “homeostasis–pathology” dichotomy. Under homeostatic or developmental conditions, knockdown of KDM6B in chondrocytes leads to abnormal cartilage development and accelerates OA progression [177]. In contrast, under OA or stress conditions, multiple studies have shown that JMJD3 upregulation is associated with cartilage destruction, and that inhibition of JMJD3—either pharmacologically (e.g., GSK‐J4) or via gene silencing—attenuates inflammation, matrix degradation, and mechanically induced OA damage [189, 216]. For LSD1, existing evidence similarly supports a strong degree of context dependence. Durand et al. reported elevated LSD1 expression in OA cartilage, accompanied by repression of matrix‐related genes such as COL9A1 [170]. Moreover, El Mansouri et al. demonstrated that under IL‐1β stimulation, LSD1 is recruited to the mPGES‐1 promoter, enhancing its transcriptional activity and amplifying inflammatory responses [187]. Taken together, these studies support the notion that LSD1 serves predominantly to amplify inflammation and disrupt matrix metabolism in OA, and that its functions in development or homeostasis cannot be automatically translated to the disease context. By contrast, DOT1L has an outwardly protective profile in studies published to date. A number of lines of evidence connect reduced expression or diminished activity of DOT1L with parallel upregulation of Wnt/β‐catenin signaling and disease progression, whereas restoration of DOT1L activity markedly alleviates cartilage degeneration [213]. Where interpretations differ between studies, it is often in the upstream regulators of DOT1L, such as hypoxic conditions or genetic polymorphisms, which could contribute to interindividual differences [164, 165]. Overall, these seemingly contradictory data are not mutually exclusive and illustrate how context‐dependent the regulation of histone methylation in OA is. Key epigenetic enzymes should not be simply categorized as pathogenic or protective factors; rather, the roles of any given epigenetic enzyme can be viewed as continually shifting, influenced by disease stage, cell type, and inflammatory or mechanical stress.

Based on available data, histone methylation in OA does not appear to confer its effects uniformly across all tissues and cell types, but rather promotes strikingly cell type‐specific facets of epigenetic regulation. In chondrocytes, this modification is primarily responsible for the maintenance of differentiation status, promotion of hypertrophic phenotypes, and regulation of transcriptional programs of ECM synthesis and degradation, resulting in epigenetic consequences most relevant to cartilage homeostasis. In synovial cells, this form of regulation appears to be more associated with the control of inflammatory response thresholds, responses to Wnt signaling, and compartment‐specific functions. In contrast, in cells associated with subchondral bone, histone methylation appears more tuned to regulating the coupling of bone remodeling with vascular and neural remodeling, as well as the structural responsiveness of the osteochondral unit to mechanical loading. Owing to inherent differences in the repertoire of transcription factors, chromatin accessibility, and the microenvironment in which the cells reside, the same histone methylation mark or regulatory enzyme may underlie entirely different biological outcomes—or even contradictory ones—across different cell types. Therefore, an accurate understanding of the functional roles of histone methylation requires a framework that explicitly accounts for cell type‐specific contexts and pathological processes.

### 5.3 Histone Acetylation

Histone acetylation refers to a reversible process in which histone acetyltransferases add acetyl groups to lysine residues on histones H3 and H4, while histone deacetylases (HDACs) or sirtuins remove these modifications. This dynamic balance establishes a regulatory switch between open and condensed chromatin states [217]. In general, acetylation marks such as H3K9ac and H3K27ac are associated with high chromatin accessibility at promoters and enhancers and active transcription, whereas deacetylation promotes chromatin compaction and transcriptional repression [218].

Work centered on HDAC4 has largely established the acetylation–chondrocyte hypertrophy axis in OA. Early studies by Lu et al. comparing normal and OA human articular cartilage demonstrated aberrant expression and subcellular localization of HDAC4 in OA cartilage; HDAC4 overexpression promoted the expression of hypertrophy‐ and matrix‐degradation‐related genes such as MMP13 and COL10A1, whereas HDAC4 silencing partially restored cartilage matrix homeostasis [219]. Subsequently, Cao further confirmed in human knee cartilage specimens that HDAC4 downregulation was strongly correlated with higher Mankin scores. Mechanistically, reduced HDAC4 relieved repression of RUNX2, resulting in sustained activation of RUNX2‐driven hypertrophic transcriptional programs [220]. More recently, Dong reported in an age‐related OA cohort that HDAC4 levels in articular cartilage declined progressively with aging and disease severity and positively correlated with the hypertrophic zone thickness, further supporting the concept that HDAC4 regulates chondrocyte hypertrophic differentiation and contributes to OA initiation [221]. In inflammatory models, Ning showed that HDAC4 knockdown markedly aggravated matrix degradation in IL‐1β‐stimulated human chondrocytes, whereas HDAC4 overexpression suppressed IL‐1β‐induced MMP13 and ADAMTS5 expression, suggesting that HDAC4 may exhibit context‐ and stage‐dependent double‐edged sword effects under different stimuli and disease phases [222].

Other HDAC members also function in modulating inflammation and stress responses that promote OA. For example, Barter showed that HDAC6 is important for properly regulating NF‐κB signaling: inhibition reduced IL‐1β–induced MMP1 and MMP13 expression and protected against cartilage explant matrix loss. However, after HDAC6 overexpression, NF‐κB activity and inflammatory and catabolic programs were enhanced [223]. These findings support those of Saito, who mechanically stressed human chondrocytes and observed an overall reduction in phosphorylation of ERK1/2, p38, and JNK when the cells were cultured in conjunction with broad‐spectrum HDAC inhibitors. In the same experiment, markers for MMP1 and MMP13 were also reduced compared with controls on average [224]. Subsequently, Gao identified natural small molecules such as obacunone that directly bind to HDAC1 to inhibit its deacetylase activity, preventing subsequent activation of p38 MAPK. In IL‐1β‐treated chondrocytes or destabilized OA mouse models, obacunone treatment improved ECM degradation and pain‐related behaviors, providing experimental evidence that selective HDAC1 inhibition may represent a potential therapeutic strategy [225].

NAD^+^‐dependent deacetylases SIRT1 and SIRT6 mechanistically link histone deacetylation to metabolism and aging across multiple dimensions. Sacitharan et al. directly demonstrated in human chondrocytes that SIRT1 promotes autophagy by deacetylating key autophagy‐related transcription factors, thereby maintaining basal autophagic flux; inhibition or knockdown of SIRT1 resulted in reduced microtubule‐associated protein 1 light chain 3‐II (LC3‐II) levels, impaired autophagy, and increased susceptibility of chondrocytes to oxidative stress and inflammatory insults, whereas SIRT1 overexpression significantly reduced chondrocyte apoptosis [226]. In OA mouse models, Lu further showed that restoration of SIRT1 activity improved articular cartilage structure and gait patterns by downregulating PTEN, suppressing epidermal growth factor receptor (EGFR) ubiquitination, and enhancing EGFR–AKT signaling and autophagy [227]. From a lipid metabolism perspective, Papageorgiou reported that SIRT1 deficiency disrupted lipid droplet homeostasis and autophagic balance, whereas SIRT1 activation restored lipophagy and reduced lipotoxicity in OA chondrocytes [228]. At the pharmacological level, Liang et al. demonstrated that resveratrol attenuated IL‐1β–induced chondrocyte inflammation and mitochondrial damage through activation of the SIRT1/FOXO1 pathway, providing a feasible example of metabolism–acetylation‐targeted intervention [229].

Regarding SIRT6, multiple studies indicate that SIRT6 expression is markedly reduced in OA cartilage. Cartilage‐specific Sirt6 knockout mice exhibit aggravated cartilage degeneration and osteophyte formation. At the cellular level, SIRT6 suppresses the IL‐15/JAK3/STAT5 axis through deacetylation, thereby reducing chondrocyte senescence and SASP factor secretion [230].Collins et al. further reported that SIRT6 activity declines significantly with aging in chondrocytes and is closely associated with accumulated DNA damage and reduced IGF‐1 signaling; restoring SIRT6 activity improved IGF‐1–AKT responses and alleviated age‐related cartilage degeneration in mice [231]. In a recent study, Mao demonstrated in IL‐1β‐stimulated OA chondrocytes that SIRT6 activation promotes DNA damage repair and reduces ROS accumulation and cellular senescence via the Keap1/Nrf2/HO‐1 pathway, whereas SIRT6 knockdown produced opposite effects [232].

### 5.4 Histone Ubiquitination

Histone ubiquitination refers to the covalent attachment of the 76‐amino acid ubiquitin protein to lysine residues on histones through an E1–E2–E3 enzymatic cascade. The two best‐characterized forms are monoubiquitination of histone H2A at lysine 119 (H2AK119ub) and histone H2B at lysine 120 (H2BK120ub1). Although this modification occurs on only a limited subset of nucleosomes, it can exert amplified transcriptional effects by altering nucleosome stability and modulating the recruitment or exclusion of reader proteins. PRC1‐catalyzed H2AK119ub acts in concert with PRC2‐mediated H3K27me3 to maintain repression of developmental and inflammatory genes, whereas H2BK120ub1, deposited by the ring finger protein 20/ring finger protein 40 (RNF20/40)–WW domain‐containing adaptor with coiled‐coil (WAC) axis, is positively associated with transcriptional elongation and H3K4/H3K79 methylation and is therefore regarded as an activating epigenetic mark [233, 234, 235, 236].

In recent years, direct evidence linking histone H2A/H2B ubiquitination to cartilage pathology in OA remains limited; however, several emerging lines of evidence have begun to outline a coherent mechanistic framework. Xu reported that monoubiquitination of histone H2B at lysine 120 (H2BK120ub1), together with its regulatory factor WAC, is significantly upregulated in cartilage from patients with RA and OA as well as in murine arthritis models, and that its levels positively correlate with the extent of matrix degradation. Inhibition of WAC reduces H2BK120ub1, restores enrichment of H3K27me3, attenuates the proinflammatory secretory phenotype of chondrocytes, and alleviates cartilage destruction in vivo. These findings suggest a functional coupling between H2B ubiquitination and H3K27 methylation, positioning this epigenetic interaction as a critical regulatory node in inflammation‐driven OA [237].

Another important line of evidence involves the H2A deubiquitinase myb‐like SWIRM and MPN domains 1 (MYSM1). MYSM1 is a well‐established H2AK119 deubiquitinase in the hematopoietic and immune systems. Wei reported that MYSM1 expression is reduced in OA cartilage, and that cartilage‐specific deletion of MYSM1 markedly exacerbates cartilage degeneration and osteophyte formation in both DMM and ACLT mouse models. Mechanistically, MYSM1 restricts sustained activation of NF‐κB and MAPK signaling by recruiting protein phosphatase 2A (PP2A) to remove K63‐linked polyubiquitin chains from receptor‐interacting serine/threonine‐protein kinase 2 and to dephosphorylate Ser176, thereby attenuating inflammatory signaling and osteochondral destruction [238].

Accumulating evidence indicates that deubiquitinases form a regulatory network linking ubiquitination dynamics with inflammation, metabolism, and cell death in OA. Ubiquitin‐specific peptidase 3 (USP3) suppresses IL‐1β‐induced NF‐κB activation by deubiquitinating TNF receptor‐associated factor 6, thereby reducing chondrocyte apoptosis and slowing OA progression in vitro and in vivo [239]. USP49, which is downregulated in OA cartilage, stabilizes axis inhibition protein via deubiquitination, promotes β‐catenin degradation, and inhibits Wnt/β‐catenin signaling, attenuating matrix degradation and cell death; small molecules such as 6‐gingerol further enhance this protective axis [240]. In contrast, USP14 is upregulated in OA and activates Wnt/β‐catenin signaling through deubiquitination of frizzled class receptor 8, thereby promoting inflammation, apoptosis, and ECM loss, whereas USP14 inhibition alleviates cartilage damage [241]. Similarly, USP25 maintains ROS‐mediated accumulation and NLRP3 inflammasome activation to induce pyroptosis and cartilage destruction; genetic silencing of USP25 significantly ameliorated structural outcomes in OA models [242]. Together with histone‐targeted DUBs such as USP16 and MYSM1, also discussed earlier, these results suggest a unifying model in which different subsets of DUBs either act directly on the ubiquitination of H2A/H2B or regulate important checkpoints via ubiquitination of chromatin (H2AK119ub/H2BK120ub), thereby promoting inflammatory and degenerative transcriptional programs in OA [243].

### 5.5 Histone Lactylation

Histone lactylation is a recently identified metabolism‐related epigenetic modification characterized by the direct coupling of intracellular lactate accumulation to chromatin‐based transcriptional regulation. Within the OA microenvironment—shaped by inflammation, hypoxia, and mechanical stress—chondrocytes and synoviocytes undergo metabolic reprogramming marked by enhanced glycolysis and increased lactate production. This shift positions the “lactate–lactylation–transcriptional reprogramming” axis as a potential mechanistic link between metabolic dysregulation and tissue degeneration in OA [244, 245, 246].

In the OA field, evidence from the past 3 years indicates that histone lactylation has progressed from a conceptual modification to a mechanistically involved regulator. Xia demonstrated in chondrocyte models and animal studies that lactate dehydrogenase A (LDHA) not only controls glycolytic flux but also enhances histone H3 lysine 18 lactylation, thereby increasing transcription of downstream metabolic genes. This LDHA‐driven H3K18la amplification reinforces the glycolytic phenotype of chondrocytes and accelerates OA progression. In vivo genetic deletion or pharmacological inhibition of LDHA mitigates cartilage damage in OA mice, supporting a pathogenic “LDHA–H3K18la–metabolic gene” regulatory axis [247, 248].

Beyond histones, lactylation in OA also modifies nonhistone proteins and interacts with canonical signaling pathways to indirectly modulate chromatin regulatory networks. Lan et al. reported increased lactate levels and pan‐lactylation in synovial fluid from OA patients and in IL‐1β‐treated primary chondrocytes. They found that lactylation of UDP‐glucose 6‐dehydrogenase impairs its enzymatic activity, interfering with glycosaminoglycan synthesis and leading to derepression of MAPK kinase kinase 8 transcription, thereby activating MAPK signaling. Inhibiting lactylation with the p300 inhibitor A485 reduces lactylation levels and markedly restores ECM homeostasis while alleviating OA progression, indicating that this modification is therapeutically targetable through a druggable acyltransferase‐mediated lactylation pathway [249]. In addition, Qian demonstrated in drug delivery and tissue repair studies that modulation of chondrocyte glycolysis and lactate production alters histone lactylation levels, thereby reshaping the transcriptional output of inflammation‐related target genes. These findings suggest that targeting the metabolic microenvironment–histone lactylation–inflammatory transcription axis may represent a feasible and actionable strategy in engineered therapeutic approaches [250].

In sum, histone lactylation provides a more unifying concept with which to understand OA pathogenesis: lactate is no longer merely a waste product of metabolism but can inscribe metabolic stress into chromatin via site‐specific lactylation, forming persistent transcriptional reprogramming that chains inflammation, metabolic dysregulation, and cell fate imbalance into a positive feedback loop. In determining the importance of this modification, the more relevant task is not to mark elevated levels of H3K18la but to interrogate whether lactylation has stage‐dependent roles (early adaptive versus late locking pathology), whether it possesses defined cell‐type and spatial specificity (cartilage versus synovium, osteochondral units, load‐bearing regions), and whether it generates a causal chain linking metabolic flux, sites of lactylation, relevant target gene regulation, and tissue‐level phenotypes. Only by addressing these issues can one delineate the translational window and safety frontiers of attempting to target the lactate–lactate dehydrogenase axis or lactylation writers such as p300 as an OA therapy.

### 5.6 Histone Serotonylation

Histone serotonylation is a recently identified epigenetic modification belonging to the class of monoaminylation. In this process, serotonin (5‐HT) is covalently conjugated to specific residues on the N‐terminal tail of histone H3—most classically at glutamine 5 (H3Q5)—through the catalytic activity of enzymes such as transglutaminase 2 (TG2/TGM2). Histone serotonylation can act in combination with activating marks such as H3K4me3 to enhance transcriptional permissiveness and thereby modulate gene expression output [251, 252, 253]. Traditionally regarded as a cytoplasmic or extracellular enzyme, TG2 also possesses a nuclear epigenetic regulatory role, enabling the integration of cellular stress and monoamine metabolic signals into chromatin state alterations. This function provides a new regulatory framework for understanding chronic inflammation‐ and tissue remodeling‐associated diseases [254].

In the field of OA research, direct evidence for histone H3Q5 serotonylation within joint tissues remains limited. However, accumulating experimental data support a pathogenic role for its key catalytic enzyme, TG2, in OA. Li et al. reported significant upregulation of TG2 in degenerative joint tissues in a temporomandibular joint OA animal model. Pharmacological inhibition of TG2 markedly ameliorated structural damage in cartilage and subchondral bone and suppressed activation of NF‐κB signaling, along with reduced expression of downstream inflammatory and matrix‐degrading mediators, including COX‐2, iNOS, MMP13, and MMP3 [255].

It should be noted that, to date, direct experimental evidence for histone serotonylation itself in OA remains lacking. This includes the absence of data on the localization and distribution of H3Q5ser or H3K4me3Q5ser in OA chondrocytes or synoviocytes, their causal relationship with TG2 activity, and whether these modifications are enriched at regulatory regions of key OA‐associated genes. Accordingly, future studies are required to systematically elucidate the authentic role of histone serotonylation in OA pathogenesis and to define its potential translational relevance.

### 5.7 Noncoding RNAs

ncRNAs comprise a broad class of regulatory RNA transcripts that do not encode proteins, including miRNAs, lncRNAs, and circRNAs. Although only approximately 2% of the human genome encodes proteins, more than 60% of the transcriptome consists of ncRNAs. Through interactions with DNA, chromatin, mRNAs, and proteins, ncRNAs exert multilayered control over gene expression and are therefore regarded as a third layer of epigenetic regulation, alongside DNA methylation and histone modifications [256, 257]. In recent years, ncRNAs have been firmly incorporated into the epigenetic regulatory framework. On one hand, miRNAs function as canonical posttranscriptional regulators by inducing target mRNA degradation or translational repression through sequence complementarity. On the other hand, lncRNAs act as molecular scaffolds or guides, recruiting enzymatic complexes such as DNMTs, EZH2, and KAT6A to specific genomic loci to remodel local chromatin accessibility and histone modification landscapes; they can also associate with transcription factors and the transcriptional machinery to directly regulate transcription initiation. CircRNAs primarily function as competing endogenous RNAs (ceRNAs) that sponge miRNAs or form complexes with RNA‐binding proteins, thereby modulating mRNA stability and alternative splicing [258, 259].

Among OA‐associated miRNAs, recent studies increasingly emphasize the dynamic balance between protective and pathogenic signals. Chen reported that miR‐140‐5p is markedly downregulated in cartilage progenitor cells (CPCs) from patients with knee OA. Restoration of miR‐140‐5p corrects IL‐1β‐induced fate dysregulation by suppressing the Jagged1/Notch signaling axis, thereby preserving chondrogenic lineage stability in CPCs and attenuating cartilage degeneration in the ACLT rat model [260]. In another animal study, Liu et al. intra‐articularly administered exosomes derived from human urine‐derived stem cells overexpressing miR‐140‐5p, which significantly suppressed VEGFA expression, reduced synovial angiogenesis, and attenuated cartilage structural damage. These findings suggest that miR‐140‐5p‐enriched exosomes may represent a promising disease‐modifying therapeutic strategy for OA [261]. In contrast, Endisha et al. demonstrated that miR‐34a‐5p is markedly elevated in the plasma, cartilage, and synovium of patients with late‐stage knee OA. In both DMM and high‐fat diet‐combined DMM mouse models, local administration of miR‐34a‐5p mimics exacerbated cartilage destruction, whereas antisense oligonucleotide‐mediated inhibition of miR‐34a‐5p improved joint structural outcomes. These findings identify miR‐34a‐5p as a pathogenic miRNA and a potential therapeutic target in OA [262]. In addition, Ji et al. reported that miR‐182‐5p levels are reduced in synovial fluid‐derived exosomes from OA patients. Restoration of miR‐182‐5p alleviates chondrocyte inflammation and apoptosis by suppressing TNF alpha‐induced protein 8 and activating LC3‐associated autophagy pathways [263].

lncRNAs and circRNAs contribute to OA epigenetic regulation largely through ceRNA networks and signaling crosstalk. Zhang et al. reported that the lncRNA small nucleolar RNA host gene 15 (SNHG15) is upregulated in OA cartilage and enhances mitochondrial antiapoptotic capacity via the SNHG15–miR‐141‐3p–BCL2 like 13 (BCL2L13) axis. In vivo, SNHG15 knockdown exacerbates cartilage matrix loss, whereas SNHG15 overexpression partially reverses OA phenotypes, suggesting that SNHG15 exhibits chondroprotective properties [264]. In contrast, Zhao et al. reported that lncRNA AFAP1–AS1 is markedly upregulated in OA cartilage and cultured chondrocytes. By sponging miR‐512‐3p, AFAP1–AS1 upregulates MMP13, promoting chondrocyte proliferation while exacerbating ECM degradation. Silencing actin filament‐associated protein 1 antisense RNA 1 (AFAP1–AS1) markedly attenuates IL‐1β‐induced ECM damage [265]. The authors further demonstrated in a clinical cohort that lncRNA Fer‐1 like family member 4 (FER1L4) is persistently downregulated in the plasma and synovial fluid of OA patients, with its levels negatively correlated with age and Kellgren–Lawrence grade receiver operating characteristic curve. In vitro, FER1L4 overexpression suppresses IL‐6 expression in chondrocytes, suggesting that FER1L4 may function both as an upstream regulator of inflammation and as a potential circulating biomarker for OA [266]. At the circRNA level, Chen demonstrated that circRNA‐9119 is downregulated in IL‐1β‐treated chondrocytes. Overexpression of circRNA‐9119 suppresses PI3K–AKT activation via the circRNA‐9119–miR‐26a–PTEN axis, thereby reducing apoptosis and promoting ECM synthesis [267]. Similarly, Wahafu et al. reported that circ_0005526 is markedly upregulated in OA cartilage tissues and IL‐1β‐stimulated chondrocytes. Silencing circ_0005526 alleviates inflammatory injury and ECM degradation by releasing its sponge effect on miR‐142‐5p, thereby downregulating the transcription factor TCF4 [268]. Another study reported that circADAMTS6 is downregulated in OA cartilage and in IL‐1β‐treated human chondrocytes. Its overexpression restores PI3K–AKT–mTOR‐dependent proliferative and matrix synthetic capacity via the circADAMTS6–miR‐324‐5p–PI3K regulatory subunit 3 (PIK3R3) axis, while reducing apoptosis and inflammatory responses [269]. Overall, miRNAs, lncRNAs, and circRNAs constitute a multilayered ncRNA regulatory network in OA. This network interfaces with canonical pathways such as Notch and PI3K–AKT–mTOR, and modulates inflammatory cytokines (e.g., IL‐6), matrix‐degrading enzymes (e.g., MMP13), and cell fate programs. Accordingly, ncRNAs act as key effectors of epigenetic reprogramming in OA, complementing DNA methylation and histone modifications (Table 3).

**TABLE 3:** Overview of OA‐associated noncoding RNAs (miRNAs, lncRNAs, and circRNAs) and their targets/pathways.

Type	Representative molecule	Primary targets/signaling pathways	Description	References
miRNA	miR‐140	ADAMTS‐5	Deficiency results in aberrant upregulation of ADAMTS‐5, thereby promoting age‐related ECM degradation and cartilage degeneration.	[270]
miRNA	miR‐140	TLR4/IL‐1β	It suppresses TLR4/IL‐1β‐induced MMP activation and restores COL2A1 and ACAN expression.	[271]
miRNA	miR‐146a	Inflammation‐related signaling pathways	Downregulated in late‐stage OA, leading to attenuation of the negative feedback regulation of proinflammatory cytokines IL‐1β and TNF‐α, thereby sustaining inflammatory responses	[272]
miRNA	miR‐146a	Camk2d/Ppp3r2	Targets Camk2d/Ppp3r2 to disrupt Ca^2^^+^ signaling and cartilage homeostasis	[273]
miRNA	miR‐146a‐5p	SDF‐1/CXCR4	Suppresses SDF‐1/CXCR4‐driven aberrant crosstalk, thereby reducing IL‐1β‐induced ECM degradation	[274]
miRNA	miR‐34a	SIRT1/p53	By inhibiting SIRT1, it enhances p53 acetylation, leading to chondrocyte apoptosis.	[275]
miRNA	miR‐34a	Apoptosis pathway	Silencing of miR‐34a significantly attenuates inflammation‐induced apoptosis and restores ECM secretion capacity.	[276]
miRNA	miR‐455	HIF‐2α	Exerts bidirectional regulation of HIF‐2α, maintaining the balance between ECM synthesis and degradation under hypoxic conditions	[277]
miRNA	miR‐17	TGF‐β/Smad	By coordinately suppressing catabolic gene expression and ECM degradation, it maintains cartilage homeostasis and alleviates mechanically induced OA.	[278]
miRNA	miR‐204	RUNX2	Activates RUNX2‐associated transcriptional programs, enhancing MMP13 and MMP3 expression and thereby promoting ECM degradation	[279]
miRNA	miR‐361‐5p	DDX20/NF‐κB	Inhibits DDX20–NF‐κB activation, thereby reducing IL‐1β‐induced inflammatory ECM degradation	[280]
miRNA	miR‐5581	NRF1/NQO1	Suppresses the NRF1–NQO1 antioxidant axis, leading to increased ROS accumulation and exacerbated cartilage inflammation and ECM damage	[281]
lncRNA	CIR	MMP13/ADAMTS5	Directly upregulates MMP13 and ADAMTS‐5 expression, leading to a marked reduction in COL2A1 levels and accelerated ECM degradation	[282]
lncRNA	CIR	miR‐27b	Acts as a sponge for miR‐27b, relieving its inhibitory effect on catabolic factors and thereby enhancing ECM degradation	[283]
lncRNA	HOTAIR	ADAMTS5	Enhances ADAMTS‐5 expression, resulting in accelerated degradation of aggrecan and other proteoglycans	[284]
lncRNA	HOTAIR	miR‐17‐5p/FUT2	Activates β‐catenin signaling via the miR‐17‐5p/FUT2 axis, thereby promoting inflammatory responses and ECM degradation	[285]
lncRNA	HOTAIR	WIF‐1	Suppresses WIF‐1 expression, leading to activation of Wnt/β‐catenin signaling and accelerated ECM breakdown	[286]
lncRNA	HOTAIR	miR‐107/CXCL12	Attenuates apoptosis through the miR‐107/CXCL12 axis while maintaining ECM synthesis in chondrocytes	[287]
lncRNA	MEG3	VEGF	Downregulates VEGF expression, suppressing inflammation‐induced angiogenesis and chondrocyte apoptosis	[288]
lncRNA	MEG3	MMP13	Suppresses MMP13 expression via the miR‐93/TGFBR2 axis, thereby restoring the balance of COL2A1 and ACAN synthesis	[289]
lncRNA	NEAT1	NEAT1/miR‐16‐5p axis	Promotes chondrocyte proliferation and inhibits apoptosis by downregulating miR‐16‐5p	[290]
lncRNA	UFC1	miR‐34a	Functions as a sponge for miR‐34a, leading to upregulation of Bcl‐2 and PCNA, thereby promoting chondrocyte survival and aberrant proliferation in OA	[291]
lncRNA	PACER	HOTAIR	Downregulation of PACER relieves its inhibitory effect on HOTAIR, resulting in enhanced apoptosis and upregulation of ECM‐degrading genes	[292]
circRNA	circSERPINE2	miR‐1271/ERG	Suppresses miR‐1271‐5p, thereby upregulating ERG, enhancing ECM synthesis, and reducing chondrocyte apoptosis	[293]
circRNA	circSERPINE2	miR‐495/TGFBR2	Attenuates IL‐1β‐induced apoptosis and MMP activation via the miR‐495/TGFBR2 axis	[294]
circRNA	circSLC7A2	miR‐4498/TIMP3	Enhances TIMP3 expression by sponging miR‐4498, thereby inhibiting MMP activity and effectively maintaining ECM integrity	[295]
circRNA	circCDK14	miR‐125a‐5p/Smad2	Upregulates Smad2 by antagonizing miR‐125a‐5p, preventing IL‐1β‐induced ECM degradation	[296]
circRNA	circFOXO3	FOXO3/autophagy pathway	Promotes FOXO3‐mediated autophagic flux, thereby reducing apoptosis and enhancing ECM homeostasis	[297]
circRNA	circGCN1L1	miR‐330‐3p/TNF‐α	Elevates TNF‐α expression via NF‐κB activation, leading to synovial hyperplasia and increased MMP13/MMP3 production, thereby promoting joint destruction	[298]

Abbreviations: CXCR4, C–X–C chemokine receptor Type 4; DDX20, DEAD‐box helicase 20; HIF‐2α, hypoxia‐inducible factor 2 alpha; TGF‐β, transforming growth factor beta; TLR4, Toll‐like receptor 4.

## 6 Therapeutic Strategies in OA

Therapy for OA encompasses a continuum of approaches, ranging from conservative management to regenerative medicine. The current treatment landscape mainly comprises nonpharmacological and pharmacological treatments of OA, aiming to relieve pain, restore joint function, and potentially alter the course of the disease. DMOADs additionally seek symptomatic relief but also target the molecular and tissue levels of the underlying biological dysfunction of OA. Simultaneously, regenerative medicine and tissue repair strategies have the potential to repair and restore joint structure and function. Surgical options remain for patients at an advanced stage of OA. Here, we outline these therapeutic modalities.

### 6.1 Nonpharmacological Treatment

Nonpharmacological interventions are widely regarded as the cornerstone and first‐line strategy for OA management throughout the disease course, while pharmacological and surgical therapies are typically introduced as adjunctive or step‐up options, a treatment framework consistently emphasized in major international guidelines. The core nonpharmacological interventions most consistently and strongly recommended across high‐quality guidelines are patient education, exercise therapy, and weight management [299, 300] (Table 4).

**TABLE 4:** Clinical studies of nonpharmacological interventions in OA.

Population and sample size	Intervention	Control	Follow‐up	Main outcomes (concise)	References
Knee OA, *n* = 206	Web‐based strengthening + PA program + automated SMS	Educational website only	24 weeks	Significant improvements in pain and function; more patients achieved MCID compared with control	[317]
Knee OA, *n* = 105	Internet‐based exercise therapy (education + structured exercises)	Usual self‐management	6 weeks	Short‐term improvements in pain and function superior to usual care	[318]
Overweight/obese knee OA, *n* = 415	Telehealth PT‐guided exercise ± structured dietary weight‐loss program	Online education only	6 + 6 months	Exercise program improved pain/function; combined diet–exercise yielded greater weight loss and slightly better symptom relief.	[319]
Knee OA, *n* = 178	Online Tai Chi program (12‐week Yang‐style videos + education + adherence app)	Education website	12 weeks	Greater pain reduction (NRS), better WOMAC function; higher MCID achievement rate.	[320]
Knee OA, *n* = 117	Yoga program (supervised + home practice)	Standardized strength‐training program	12//24 weeks	Yoga noninferior for pain at 12 weeks; better WOMAC and QoL outcomes by 24 weeks	[321]
Moderate–severe knee OA, *n* ≈ 195	mHealth sensor‐guided self‐exercise program	Usual care	12 weeks	Significant KOOS pain improvement; small–moderate effect size; good feasibility	[322]
Medial knee OA, *n* = 120	Custom off‐loader knee brace + standard care	Standard care	12 months	Greater pain reduction; KOOS improvements across domains; reduced analgesic use; cost effective	[323]

Abbreviations: KOOS, Knee injury and Osteoarthritis Outcome Score; MCID, minimal clinically important difference; NRS, Numerical Rating Scale; PA, physical activity; PT, physical therapy; QoL, quality of life; SMS, short message service.

#### 6.1.1 Patient Education and Self‐Management

Patient education aims to equip individuals with knowledge about the disease, prognosis, and practical behavioral strategies, including pain coping skills, joint protection, and exercise prescriptions. All high‐quality guidelines explicitly recommend education and self‐management programs as foundational interventions, ranking them alongside exercise and weight management as core treatments for OA [299]. The nonpharmacological treatment recommendations issued by European League Against Rheumatism further emphasize that combining structured education programs with exercise therapy yields greater pain relief and functional improvement than exercise alone, and helps enhance long‐term adherence [301]. Common delivery formats include face‐to‐face group sessions, individualized education, telephone‐ or web‐based follow‐up, and self‐management programs grounded in cognitive behavioral therapy principles.

#### 6.1.2 Exercise Therapy

Exercise therapy is one of the most robustly supported nonpharmacological interventions and is strongly recommended by multiple guidelines as a first‐line treatment for knee and hip OA. Major exercise modalities include: (1) aerobic exercise (e.g., walking, cycling, aquatic aerobics); (2) resistance/strength training; (3) mind–body exercise (e.g., Tai Chi, yoga); and (4) multimodal or combined training programs. Mo et al. conducted a systematic review and network meta‐analysis of exercise interventions for knee OA, encompassing 152 randomized controlled trials (RCTs). This analysis found that a variety of exercise regimens, including aerobic exercise, resistance training, Tai Chi, yoga, and cycling, had significant positive effects on pain, stiffness, and physical function. Aerobic exercise had the largest effect on pain reduction, with yoga being more effective for improving joint function and general health measures [302]. A 2024 meta‐analysis of physical therapy and rehabilitation interventions confirmed similar findings, identifying aerobic exercise and strength training interventions as having “moderate‐certainty evidence” for pain and functional improvement, making them silver‐level recommended interventions [303].

As far as dosage and delivery are concerned, the guidelines recommend that moderate‐intensity exercise should be performed at least three times per week for 30–60 min per session for at least 8–12 weeks as one full course of treatment, and that individual prescriptions must be tailored, starting slowly and progressing gradually based on the patient's age, comorbidities, and functional status [304].

#### 6.1.3 Weight Management

Overweight and obesity are major risk factors for the onset and progression of knee OA, and weight management is another fundamental nonpharmacological intervention strongly recommended by most clinical guidelines [305]. In a recent meta‐analysis, Robson et al. found that, compared with usual care or minimal interventions, weight‐loss interventions led to small to moderate improvements in pain and physical function. Combined interventions (diet + exercise) were more efficacious than diet alone [306]. Several studies and clinical practice guidelines suggest that, for overweight or obese patients with OA, a 5–10% reduction in body weight is sufficient to achieve clinically meaningful improvements in pain and physical function; greater weight loss leads to greater reductions in joint load and consequently greater improvements in symptoms [307, 308]. Thus, for overweight or obese patients with OA, structured weight‐management programs should be initiated early in the disease course. Weight management should include calorie‐controlled dietary approaches, behavioral interventions, and individualized exercise prescriptions. Whenever feasible, a multidisciplinary team (e.g., dietitians, physiotherapists, and psychologists) should oversee care so that patient‐preferred dietary approaches can be incorporated to improve adherence and clinical outcomes.

#### 6.1.4 Biomechanical Interventions

To relieve symptoms and potentially slow the progression of structural damage, biomechanical interventions aim to improve load distribution across joints and reduce mechanical loading. Biomechanical strategies can include lower‐limb orthotic devices or braces, including knee braces (particularly valgus braces for medial‐compartment OA) and lower‐extremity orthotic devices or shock‐absorbing insoles, which help reduce load through the affected compartment, improve pain, and alter gait mechanics [309, 310]. The addition of assistive devices (e.g., canes, or walkers) may lessen discomfort by changing how much weight is placed on the injured joint, redistributing load to other parts of the body so that less strain is placed on the joint. This, in turn, may also help reduce the risk of falling in older adults and in other individuals with a higher likelihood of falls [311]. The evidence supporting the use of insoles and knee braces to relieve pain or improve function can be described as being of low to moderate quality. Patients with marked varus deformity show greater benefit from the use of insoles and knee braces than patients with better alignment. Therefore, both of these interventions should be provided only as optional or conditional recommendations and should be offered to individuals who have already received first‐line nonpharmacological treatment [312, 313].

#### 6.1.5 Physical Modalities and Rehabilitation

In recent years, several real‐world clinical studies have evaluated the role of physical therapy modalities in OA management. Regarding electrical stimulation, the ETRELKA RCT compared transcutaneous electrical nerve stimulation (TENS) with sham stimulation in patients with knee OA and found that, at 6 weeks, TENS did not provide superior improvements in pain or function compared with the control group, suggesting limited therapeutic benefit [314].

However, in another double‐blind randomized trial, TENS combined with a home‐based exercise program significantly reduced pain and increased quadriceps strength after 12 weeks, suggesting that TENS may exert synergistic effects when integrated with specific cointerventions [315]. For extracorporeal shock wave therapy (ESWT), a 2024 systematic review and meta‐analysis reported that ESWT significantly improves pain (VAS), WOMAC functional scores, and range of motion in patients with knee OA [316]. These studies suggest that physical modality therapies are generally safe and may provide benefits in selected patients; however, treatment effects vary substantially across modalities, and high‐quality trials with long‐term follow‐up remain limited. Accordingly, physical modalities are better positioned as adjuncts to exercise therapy and lifestyle management, to be selectively applied by rehabilitation teams based on individual functional status and treatment tolerance.

### 6.2 Pharmacologic Therapies

Currently, there is no approved or universally recognized pharmacological agent as a definitive DMOAD. Therefore, the principal functions of pharmacotherapy are to provide pain relief and improve function while reducing the risk of adverse events and comorbidities during the long‐term treatment of OA. The most up‐to‐date high‐quality guidelines and consensus documents, including the 2019 ACR/AF guidelines and recommendations from Osteoarthritis Research Society International (OARSI) and ESCEO published since then, support the use of pharmacotherapy in conjunction with core treatments (exercise, weight loss, and education) in a stepwise, individualized approach, with local treatments first, followed by oral medications if necessary, and the use of injections when appropriate [300, 324].

NSAIDs are the predominant pharmacological class for managing symptomatic (moderate to severe) OA pain. A network meta‐analysis of 74 RCTs indicated that etoricoxib 60 mg/day and diclofenac 150 mg/day rank among the most effective oral NSAIDs (for both pain relief and functional improvement) within standard dose ranges; however, both were associated with a slightly increased incidence of adverse events. Patients who are at high risk for serious cardiovascular or GI complications, or who require long‐term NSAID treatment, may otherwise benefit from these potent NSAIDs, but should avoid using either agent due to their increased adverse events profiles [325]. Accordingly, guidelines emphasize that when oral NSAIDs are indicated, clinicians should use the lowest effective dose for the shortest duration, carefully assess cardiovascular, GI, and renal risks, and consider gastroprotection (e.g., proton pump inhibitors) when appropriate [300, 305].

Given that many OA‐affected joints—particularly the knee and hand—are relatively superficial, topical NSAIDs have gained increasing prominence in recent studies and clinical guidelines. A 2020 meta‐analysis of RCTs including five studies found that, compared with vehicle control, topical diclofenac solution produced statistically significant improvements in WOMAC pain, stiffness, and function scores, as well as walking pain. Rates of GI and systemic adverse events were comparable between groups, although mild local reactions, such as skin dryness or irritation were slightly more frequent [326]. Accordingly, multiple clinical guidelines—including those from OARSI—recommend topical NSAIDs as a first‐line pharmacological option for knee OA, particularly in older patients with multiple comorbidities or those receiving antiplatelet or anticoagulant therapy [324].

For patients with features suggestive of central sensitization, comorbid depression or anxiety, or an inadequate response to NSAIDs, the adjunctive use of centrally acting analgesics may be considered, with duloxetine supported by relatively robust evidence. In a 13‐week randomized, double‐blind, placebo‐controlled Phase III trial conducted by Uchio et al., duloxetine significantly reduced pain scores and improved WOMAC function and quality of life in patients with knee OA. No significant change in radiographic joint space width was observed. Adverse events were mainly nausea, dry mouth, and fatigue, and the overall safety profile was consistent with its established indications [327]. Subsequent randomized, double‐blind studies conducted in Chinese patient populations have reported largely consistent results [328]. Accordingly, the American College of Rheumatology/Arthritis Foundation (ACR/AF) guideline issues a conditional recommendation for duloxetine in knee OA, particularly in patients with features of central sensitization or comorbid mood disorders, and does not recommend its use as a first‐line therapy for all OA patients [300, 305].

Symptomatic slow‐acting drugs for OA (SYSADOAs)—including glucosamine, chondroitin sulfate, and plant‐derived combination preparations—remain among the most debated pharmacological categories. RCTs such as the CONCEPT study have shown that pharmaceutical‐grade chondroitin sulfate (800 mg/day) provides significant improvements in pain and function compared with placebo over 6 months, with efficacy comparable to that of celecoxib for certain outcomes [329]. An analysis of SYSADOA‐related RCTs across Eastern and Western regions reported that glucosamine, chondroitin sulfate, and plant‐derived preparations (e.g., SKCPT/SKI306X) were superior to placebo for improving pain and function over 12 months, and in several studies their efficacy was noninferior to NSAIDs with a favorable safety profile [330]. However, interpretation of the same evidence base differs substantially across guidelines. European Society for Clinical and Economic Aspects of Osteoporosis, OA and Musculoskeletal Diseases positions pharmaceutical‐grade crystalline glucosamine and long‐acting chondroitin sulfate as early options within its stepwise pharmacological algorithm for knee OA. In contrast, OA Research Society International and several other international guidelines, citing limited evidence quality and small effect sizes, strongly recommend against the routine use of glucosamine and chondroitin sulfate preparations in knee OA [324].

To summarize, new evidence from RCTs and meta‐analyses shows that SYSADOAs may provide approximately a 20–30% improvement in some mild‐to‐moderate knee OA symptoms while maintaining a favorable long‐term safety profile. However, considerable uncertainty remains regarding their effectiveness for specific symptoms (e.g., differences between formulations), the relative efficacy of different SYSADOAs, the consistency of treatment effects across formulations, and whether SYSADOAs represent a cost‐effective therapeutic option. This indicates that SYSADOAs should be used as adjunctive therapies and combined with other first‐line treatments for knee OA.

Diacerein is considered a distinct SYSADOA with potential structural and/or sustained symptomatic benefits. The Diacerein International Study of Safety in Osteoarthritis trial was an international, multicenter, double‐blind, noninferiority RCT comparing diacerein with celecoxib for symptomatic knee OA. Over 6 months, diacerein achieved improvements in pain and function comparable to those of celecoxib and met the prespecified noninferiority margin. However, GI adverse events—particularly diarrhea—were more frequent in the diacerein group [331]. Taken together, given that the European Society for Clinical and Economic Aspects of Osteoporosis, OA and Musculoskeletal Diseases assigns diacerein a weak recommendation, while OA Research Society International provides no specific endorsement, current evidence does not support its routine use. Diacerein may be more appropriately considered on an individualized basis, particularly for patients who are intolerant to conventional NSAIDs or have high cardiovascular risk [324].

Intra‐articular injection therapies are typically considered part of pharmacological management and mainly include intra‐articular corticosteroids (IACS), hyaluronic acid (HA), and PRP. Multiple RCTs and meta‐analyses show that IACS can provide significant pain relief in knee OA within several weeks, but the effect often wanes after 4–6 weeks. A randomized clinical trial reported that repeated intra‐articular triamcinolone injections administered every 3 months for 2 years resulted in greater cartilage volume loss compared with saline controls, without providing sustained benefits in pain relief [332]. As a result, clinical guidelines generally position IACS as a short‐term rescue therapy for patients with marked synovitis or acute pain flares, and explicitly discourage frequent or long‐term repeated intra‐articular injections.

Compared with IACS, intra‐articular HA injections have increasingly shown limited clinical benefit in long‐term practice. Evidence from a large systematic review involving 169 trials indicates that viscosupplementation provides a statistically significant but clinically modest reduction in knee OA pain compared with placebo. The analysis also found a higher relative risk of serious adverse events, and therefore did not support the widespread use of HA injections for knee OA [333]. Accordingly, the ACR/AF guideline issues a conditional recommendation against HA injections for knee OA and a strong recommendation against their use in hip OA, reserving HA only as a late‐line option in selected regions or patient populations [300].

PRP injections have been increasingly adopted in clinical practice over recent years; however, high‐quality trial evidence does not support their routine use. The Randomized Evaluation of Safety and Efficacy of Oral Collagen for Osteoarthritis (RESTORE) trialA was a randomized, double‐blind study including 288 patients with mild‐to‐moderate knee OA, comparing three intra‐articular PRP injections with saline. At 12‐month follow‐up, no significant between‐group differences were observed in pain scores or changes in medial tibial cartilage volume [334]. Although some small‐to‐moderate RCTs and meta‐analyses have reported that PRP may outperform HA at certain time points, substantial heterogeneity in PRP preparation, dosing regimens, and outcome measures limits comparability and prevents robust synthesis. Consequently, both the ACR/AF and OARSI guidelines strongly recommend against the routine use of PRP for knee or hip OA.

Based on clinical trials and systematic reviews published over the past 5 years, pharmacologic management of OA can be summarized as follows. Topical and oral NSAIDs remain the mainstay for symptom control. Centrally acting analgesics such as duloxetine may be added on an individualized basis for selected phenotypes (e.g., central sensitization or mood comorbidity). The efficacy and positioning of SYSADOAs remain inconsistent across studies and guidelines, and they should be used cautiously with explicit disclosure of the uncertainty in benefit. For intra‐articular therapies, corticosteroid injections are best reserved for short‐term rescue treatment, whereas HA and PRP offer limited overall benefit and raise ongoing concerns regarding safety and cost, and therefore should not be considered routine first‐line options (Table 5).

**TABLE 5:** Summary of key pharmacologic therapies for OA and major supporting evidence.

Drug class	Main clinical findings	References
Oral NSAIDs	Etoricoxib 60 mg/day and diclofenac 150 mg/day showed the largest effects on pain and function among NSAIDs, but with slightly increased adverse events.	[325]
Topical NSAIDs	Topical diclofenac significantly improved WOMAC pain, stiffness, function, and walking pain vs. vehicle; systemic adverse events similar to placebo; mild local skin reactions increased.	[326]
Central acting analgesics (e.g., duloxetine)	Duloxetine reduced knee OA pain and improved function and HRQoL; adverse events consistent with known profile; no radiographic structural worsening.	[327]
SYSADOAs (chondroitin sulfate, glucosamine, plant‐based compounds)	Pharmaceutical‐grade chondroitin sulfate improved symptoms vs. placebo and was comparable to celecoxib in some RCTs; SYSADOAs show modest benefits with good safety but high heterogeneity.	[329]
Diacerein	Noninferior to celecoxib for symptom relief at 6 months; GI adverse events (especially diarrhea) more frequent.	[331]
IACS	Short‐term pain relief lasting 2–6 weeks; repeated injections associated with accelerated cartilage loss without long‐term pain benefit.	[332]
HA injections	Pain reduction statistically significant but below minimal clinically important difference; higher risk of serious adverse events vs. placebo.	[333]
PRP	No significant improvement in pain or medial tibial cartilage volume vs. saline at 12 months.	[334]

Abbreviation: HRQoL, health‐related quality of life.

### 6.3 DMOADs: Promises and Lessons Learned From Clinical Failures

Current OA management strategies have led to significant improvements in pain relief and functional outcomes, but an increasing body of high‐quality evidence suggests that they exert limited effects on structural disease progression. Both systemic analgesics and intra‐articular injections appear to provide primarily short‐term symptomatic benefits, with limited or inconsistent efficacy in delaying degeneration of hyaline cartilage or modifying pathological processes associated with osteochondral unit homeostasis. Even for commonly used injection therapies discussed previously—such as IACS, HA, and PRP—this dissociation between symptom improvement and ongoing structural deterioration has been repeatedly observed across RCTs and long‐term follow‐up studies.

Accordingly, efforts to develop DMOADs have gained momentum. The central goal of these strategies is not more potent analgesia, but rather the intervention of key pathological processes driving OA progression, with the aim of achieving measurable and durable structural benefits—such as slowing cartilage loss and correcting aberrant remodeling of the osteochondral unit—and, ideally, translating these effects into sustained long‐term clinical improvement [335, 336]. Precisely because DMOADs aim to alter the natural course of disease, they are conceptually highly attractive; however, this goal also sets a more stringent evidentiary bar for clinical translation. Beyond demonstrating changes in imaging or structural endpoints, DMOADs must also establish a robust and reproducible link between structural modification and meaningful patient benefit.

Among candidates that have undergone more rigorous structural evaluation, recombinant human fibroblast growth factor 18 (FGF18), also known as sprifermin, represents a representative example. Five‐year follow‐up of a clinical study showed a demonstrable dose–response relationship for total femorotibial cartilage thickness, with 100 µg administered every 6 months having a sustained cartilage thickness advantage over placebo through Year 5, affording a clear signal of structural effect. However, in the same study, WOMAC pain scores improved significantly in all groups, with no consistent treatment‐by‐group differences, indicating that improvements in structure do not necessarily coincide with symptom improvement [337]. To address the critical issue of structure–symptom dissociation, investigators in that study undertook exploratory analyses within subjects with a high‐risk progression subphenotype and found that other studies may suggest structural change and symptom outcomes appear to track more closely together in selected populations. This indicates that the success of DMOADs may be highly phenotype dependent and therefore not readily transferable across all OA patients [338]. Meanwhile, elucidating pain pathways highlights a different translational hurdle. In randomized trials comparing with NSAIDs, tanezumab (an NGF inhibitor) produced statistically significant improvements in pain and functional outcomes; however, it was associated with a higher, dose‐dependent rate of joint safety events, including structure‐related adverse outcomes. As a result, the overall risk–benefit profile has been difficult to reconcile with the clinical requirements of a disease‐modifying therapy [339, 340].

Behind this apparent difficulty in closing the signal loop, there are broader systemic challenges arising from trial design and disease heterogeneity. For example, in the Phase IIb randomized trial of the Wnt pathway modulator lorecivivint, treatment effects at 24 weeks were primarily reflected in patient‐reported outcomes and were used to inform dose selection for subsequent studies. However, the short study duration and the relatively slow structural progression of the enrolled population limited the ability to robustly evaluate structural endpoints [341]. Accordingly, experience over the past 5 years increasingly supports the view that repeated setbacks in DMOAD development do not reflect the failure of a single drug or pathway. Rather, they are constrained by the multitissue pathology and pronounced heterogeneity of OA, as well as by current trial frameworks that remain insufficient in participant enrichment, combined endpoint strategies (structure plus symptoms), and follow‐up design—to reliably capture the integrated effects of true disease modification.

### 6.4 Regenerative Medicine and Tissue Engineering

#### 6.4.1 MSCs

In recent years, MSCs have become one of the most clinically advanced cell‐based strategies in regenerative medicine for OA. MSCs are adult stem cells with multilineage differentiation capacity and prominent immunomodulatory properties, and they are widely distributed in tissues such as bone marrow, adipose tissue, synovium, and umbilical cord. Evidence suggests that MSCs exhibit limited survival after intra‐articular administration, and their therapeutic effects in OA are mediated primarily through paracrine mechanisms, including anti‐inflammatory cytokines, growth factors, and exosome‐mediated signaling. Through these networks, MSCs may suppress synovial inflammation, reduce cartilage matrix degradation, and improve homeostasis within the joint microenvironment [342, 343]. In recent years, clinical studies have primarily focused on intra‐articular MSC injections for knee OA, evaluating autologous or allogeneic adipose‐derived MSCs and different cell preparations for their effects on pain and functional outcomes. Available evidence suggests that, in certain study designs and patient populations, MSC therapy may be associated with symptomatic improvement compared with placebo, whereas other rigorously conducted double‐blind controlled trials have failed to demonstrate significant differences (Table 6).

**TABLE 6:** Recent clinical trials of cell‐based therapies for knee OA (last 5 years).

Design and population	Intervention vs. control	Primary endpoint and follow‐up	Key clinical findings	Structural outcomes and safety	Study (year)
Multicenter, double‐blind; K–L Grade 3 knee OA (*n* = 261)	Single intra‐articular injection of autologous expanded adipose‐derived MSCs vs. placebo	VAS pain and WOMAC at 6 months	Greater improvement in VAS (−25.2 vs. −15.5 mm) and WOMAC (−21.7 vs −14.3) compared with placebo	No significant between‐group difference in MRI cartilage defects; no treatment‐related serious adverse events	[344]
Phase IIb, multicenter, double‐blind RCT; mild–moderate knee OA (*n* = 97)	Single injection of autologous adipose‐derived MSCs (2 × 10^6^ or 10 × 10^6^) vs. saline	OARSI/OMERACT strict responder rate at 6 months	No superiority over placebo (47.3 vs. 54.8% responders; RR 0.86, *p* = 0.46)	No significant differences in MRI cartilage thickness or secondary outcomes	[345]
Double‐blind, placebo‐controlled RCT; K–L Grade 2–3 knee OA (*n* = 120)	Microfragmented adipose tissue (MFAT) vs. saline	KOOS4 at 6 months (follow‐up to 24 months)	MFAT not superior to placebo at any time point; both groups showed clinically relevant improvement	Authors conclude lack of efficacy beyond placebo; no major safety signals.	[346]
Multicenter randomized trial; K–L II–IV knee OA (*n* = 480)	BMAC, UCT‐MSCs, or SVF vs. corticosteroid injection	VAS pain and KOOS pain at 12 months	None of the biologic treatments superior to corticosteroids; no optimal cell source identified	No meaningful structural improvement on imaging; favorable safety profile	[347]
Phase I/II randomized, active‐controlled trial	Allogeneic adipose‐derived MSC product vs HA	WOMAC, VAS up to 48 weeks	Early and sustained improvements in pain and function suggested, exploratory efficacy	Primarily a safety and feasibility study; requires confirmation in larger placebo‐controlled trials	[348]

Abbreviations: BMAC, bone marrow aspirate concentrate; K–L, Kellgren–Lawrence; KOOS4, aggregate score of four Knee injury and Osteoarthritis Outcome Score subscales; SVF, stromal vascular fraction; UCT‐MSCs, umbilical cord tissue‐derived mesenchymal stem cells; VAS, visual analog scale.

#### 6.4.2 Exosome‐Based Therapies

Exosomes are small extracellular vesicles, typically measuring 30–150 nm in diameter, that carry bioactive cargo such as miRNAs, proteins, and lipids. They mediate intercellular communication and can remodel the joint microenvironment by transferring regulatory signals between cells [349]. Compared with cell‐based therapies, exosomes, as cell‐free products—are theoretically more amenable to standardized manufacturing, storage, and delivery. They are also considered more compatible with biomaterial‐based systems, which may prolong intra‐articular retention and enhance targeting efficiency [350, 351]. Recent studies have increasingly focused on two key questions. First, can exosomes reprogram chondrocytes from a catabolic to an anabolic state in inflammatory and degenerative environments? Second, can bioengineering strategies be used to achieve enhanced cartilage targeting and more clearly defined molecular mechanisms? For example, Liu developed dual‐engineered extracellular vesicles derived from umbilical cord MSCs: collagen Type II‐binding peptides were displayed on the vesicle surface to enhance cartilage targeting, while miR‐223 was loaded as a therapeutic cargo. These vesicles alleviated OA‐related pathology by suppressing NLRP3 inflammasome activation and chondrocyte pyroptosis, illustrating the potential of engineered exosomes for mechanism‐based cartilage protection [351]. With respect to the immune microenvironment, Li showed in a rat OA model that bone marrow MSC–derived exosomes reduce inflammation and cartilage damage by modulating synovial macrophage M1/M2 polarization, a process associated with PINK1/Parkin signaling. Building on this, Sun engineered BMP‐7‐modified exosomes and demonstrated that they alleviate knee OA‐associated inflammation and cartilage injury by promoting macrophage polarization toward the M2 phenotype. Collectively, these studies reinforce a reproducible mechanistic axis linking exosomes, macrophage polarization, and cartilage catabolism [352, 353].

Clinical translatability represents one aspect in which trials involving exosomes have been conducted in a rigorous manner. In an early‐phase randomized, double‐blind, dose‐escalation clinical trial evaluating intra‐articular injection of umbilical cord‐derived MSC‐derived exosomes in knee OA, Wang et al. assessed safety as the primary outcome and subsequently performed exploratory assessments of pain scores, functional outcomes, and MRI parameters. The treatment was well tolerated, and during the follow‐up period, improvement trends were observed in selected clinical scores and imaging parameters [350].

#### 6.4.3 Biomaterials

In OA, tissue engineering and biomaterial approaches aim to create controllable local microenvironments in joint cavities or lesions rather than simply replacing or repairing damaged or lost tissue. These approaches allow the simultaneous modulation of multiple factors to influence pain, inflammation, cartilage matrix degradation, and regenerative processes within a predictable time frame. Compared with typical intra‐articular drug injection treatments, these techniques place significantly greater emphasis on understanding the relationships among material design, the pathological microenvironment of the joint, and how these interacting components ultimately influence biological activity (including the local residence time of therapeutics, control of release kinetics, and the bioactive properties of the biomaterials).

In OA drug delivery research, it is not sufficient to consider hydrogels or microspheres simply as passive reservoirs for drug delivery. The greater objective is to design systems in which the structural properties of the material and drug release kinetics are actively integrated with key pathological signals in the OA joint. For example, an injectable system that forms a gel or particles within the joint cavity can create a three‐dimensional matrix that enables prolonged local retention and an extended drug delivery window, providing a stable intra‐articular delivery platform for nucleic acids, proteins or antibodies, and small molecules [354]. Yu Zhang et al. created a thermoresponsive in situ‐gelling system that encapsulated exosomes derived from PRP, allowing sustained release of exosomes over 28 days. In vivo studies showed that this approach improved local retention and delayed disease progression while reducing chondrocyte apoptosis and hypertrophy. It was found that the longevity of the system primarily resulted from physical confinement of the exosomes within the postgelation matrix rather than from repeated injections over time [355]. Through the use of a self‐assembling hydrogel composed of naproxen and a peptide (NpxFFK) created by Yang, greater retention and prolonged delivery were observed for the anti‐inflammatory effects during intra‐articular administration. In addition, more extensive histological improvement and reduced inflammation were observed when comparing the NpxFFK hydrogel with either the free drug or HA as controls in a rat OA model, thus supporting self‐assembling hydrogels as effective platforms for intra‐articular drug delivery [356]. A continuing series of mechanism‐based advances has since linked release characteristics to the pathological microenvironment of OA. Using MMP overexpression as a trigger, Li et al. developed an MMP‐responsive injectable hydrogel that exhibited clear enzyme‐triggered release under simulated enzymatic conditions. In vivo, the material signal gradually declined over time (remaining detectable up to Day 28 and then disappearing), and in a rat OA model, it improved the expression profile of proteins related to the balance between ECM synthesis and degradation [357]. A similar strategy has been applied to miRNA delivery. For example, sustained release of miR‐29b‐5p via a hydrogel, combined with endogenous stem cell recruitment, has been shown in OA models to suppress matrix‐degrading enzymes and senescence‐associated genes while promoting cartilage repair‐related responses. These findings further demonstrate that coupling material design with pathological processes can transform nucleic acid therapeutics from transient exposure into verifiable histological benefits [358]. Concurrently, enzyme‐responsive microspheres have been developed to formulate anti‐inflammatory small molecules for lesion‐specific, enzyme‐triggered release. More advanced multistimuli‐responsive strategies integrate structural retention, pathology‐responsive release, and multitarget intervention to simultaneously modulate neurovascular abnormalities and pain behaviors. Recent studies have reported a pH/ROS dual‐responsive hydrogel system that scavenges extracellular RNA while enabling sustained release of bevacizumab, thereby inhibiting neurovascular ingrowth at the osteochondral interface, alleviating chronic pain, and slowing disease progression [359].

Beyond injectable delivery and sustained release, another major research direction has been the use of hydrogels or porous scaffolds as biomimetic ECM analogues. In this paradigm, the focus shifts from what to load to how the material itself regulates cells through mechanostructural cues. Here, the elastic modulus, stress–response behavior, and pore connectivity architecture are treated as programmable biophysical signals that can reshape cell adhesion, spreading, migration, and differentiation trajectories. For example, to approximate the native mechanical properties of cartilage, some studies have developed cell‐free hydrogel scaffolds with cartilage‐like mechanical strength and resistance to repeated loading and demonstrated that scaffold mechanics directly influence the cell migration, proliferation, and differentiation of nearby cells. The general strategy is first to provide a stable three‐dimensional microenvironment that recruits bone marrow‐derived mesenchymal stem/stromal cells, and then apply subsequent cues to drive chondrogenic differentiation and defect repair [360]. At a more controlled in vitro mechanistic level, Ai systematically compared the effects of the same chondroitin sulfate methacrylate/gelatin methacrylate three‐dimensional hydrogels containing cell‐adhesive motifs but with different initial Young's moduli (∼33 vs. ∼8 kPa) on MSC morphology and chondrogenic differentiation. Their results showed that matrix stiffness not only alters the morphological evolution of cellular protrusions but also modulates the expression of chondrogenic markers, indicating that tuning material stiffness toward the tissue‐relevant mechanical window can directly influence cell fate decisions [361].

Biomaterials used in cell‐free tissue engineering are designed to serve two main purposes: they facilitate the migration of host cells to areas requiring repair (homing) and support the regeneration process through coordinated structural and temporal control. Because of this, cell‐free tissue engineering avoids the high cost and technical difficulty associated with direct cell transplantation. A representative example is the work by Dong et al., who used an injectable composite biomaterial with a mesh‐like architecture that enabled the sequential release of a chemokine to promote MSC migration, followed by a chondrogenic small molecule to induce MSC differentiation in vitro, thereby supporting cartilage regeneration in an animal model with a cartilage defect [362]. Another example of a bioactive cue developed by Li et al. includes an injectable adhesive hydrogel used as a scaffold, combined with engineered microspheres derived from the chondrogenic MSC secretome to function as bioactive signaling cues [363].

It is important to emphasize that not all in situ gelling or ECM‐mimetic materials yield protective or reparative outcomes in vivo, and apparent ex vivo benefits, such as lubrication or reinforcement do not necessarily translate into meaningful in vivo efficacy. Asadikorayem reported in a collagenase‐induced rat OA model that an in situ‐forming zwitterionic hydrogel showed tissue penetration, prolonged retention, and lubricating/protective effects in cartilage explants, yet failed to prevent cartilage degeneration in vivo. More notably, under certain conditions, a non‐in situ‐crosslinked zwitterionic polymer was instead associated with more severe cartilage degradation, accompanied by increased synovial inflammation and immune cell infiltration [364].

At the level of clinical translation, OA‐related biomaterial evidence has largely evolved along two paths. One focuses on symptom‐oriented intra‐articular injectable biomaterials, whereas the other is structure‐repair‐oriented, primarily targeting patients with focal full‐thickness cartilage defects or those with early OA changes accompanying localized chondral lesions (Table 7).

**TABLE 7:** Clinical studies of intra‐articular biomaterials in OA.

Material/product (clinical pathway)	Indication	Study design	Regimen	Comparator	Follow‐up	Key outcomes	References
iPAAG; single intra‐articular injection (symptom‐oriented)	Knee OA (predominantly moderate–severe adults)	Randomized controlled, noninferiority	Single 6 mL iPAAG	HA	52 weeks	WOMAC pain: iPAAG improvement noninferior to HA; secondary outcomes broadly similar; conclusion: single iPAAG can provide symptom improvement up to 1 year.	[365]
iPAAG (prospective open‐label “real‐world” follow‐up)	Knee OA (mild–severe)	Multicenter, prospective open‐label	Single iPAAG injection	None	12 months	WOMAC pain and related outcomes showed clinically meaningful improvements through 52 weeks; authors note need for confirmatory RCTs.	[366]
PN viscosupplement/biomaterial injectate (symptom‐oriented)	Knee OA	Randomized controlled trial	PN once weekly × 3	High‐molecular‐weight HA (HMWHA), same schedule	16 weeks	PN showed comparable efficacy and safety vs. HMWHA over 16 weeks (pain/function endpoints).	[367]
HASA (human amniotic suspension allograft) intra‐articular injection (symptom‐oriented)	Unilateral knee OA	Prospective, nonrandomized pilot	Single 3 mL injection	None	3/6/12 months	IKDC and VAS significantly improved at 3, 6, and 12 months vs. baseline; conclusion: appears safe and potentially effective.	[368]
ASA (amniotic suspension allograft) intra‐articular injection (symptom‐oriented)	Symptomatic knee OA	Multicenter, randomized controlled (Level I)	Single IA injection (ASA vs. HA vs saline in the trial)	HA; saline	12 months	KOOS and VAS improvements sustained to 12 months; ∼63.2% OARSI/OMERACT responders at 12 months; no differences in immunologic markers reported.	[369]
Atelocollagen intra‐articular injection (symptom‐oriented)	Knee OA	Retrospective real‐world study	Repeated IA injections (per study protocol)	None	Per records	Pain relief associated with repeat use; authors emphasize need for higher‐quality RCT evidence.	[370]

Abbreviations: ASA, amniotic suspension allograft; IKDC, International Knee Documentation Committee; iPAAG, injectable polyacrylamide hydrogel; PN, polynucleotides.

### 6.5 Surgical Treatment

When patients with OA continue to experience persistent pain and substantial functional impairment despite adequate nonsurgical and pharmacologic management, and imaging demonstrates irreversible structural joint damage, surgical intervention may be considered. The primary goals of surgery are to relieve pain, restore function, and reconstruct the joint's biomechanical environment [371].

In patients with degenerative knee pain and/or degenerative meniscal tears associated with knee OA, the benefits of arthroscopic surgery have been markedly attenuated in high‐quality trials. Sham‐controlled randomized trials have shown that arthroscopic partial meniscectomy provides no meaningful advantage over sham surgery [372]. Subsequent randomized controlled follow‐up studies similarly failed to demonstrate sustained advantages in either symptom relief or functional outcomes [373]. In addition, 5‐year follow‐up analyses that incorporated imaging outcomes indicated partial meniscectomy was associated with an increased risk of radiographic OA progression [374]. In contrast, for patients with end‐stage knee OA who meet indications for joint replacement, the clinical benefits of surgery are more clearly supported by RCTs. One RCT demonstrated that total knee arthroplasty (TKA) combined with nonsurgical management resulted in significantly greater pain relief and functional improvement at 12 months compared with nonsurgical treatment alone [375]. Moreover, parallel randomized trials with 2‐year follow‐up similarly demonstrated greater overall improvements in the arthroplasty group compared with nonsurgical management [376].

For compartment‐specific arthroplasty in knee OA, the Warwick randomized trial provided a direct head‐to‐head comparison between patellofemoral arthroplasty (PFA) and TKA in patients with isolated patellofemoral OA. The central question of the trial was whether the two procedures differed in functional outcomes [377]. Based on cost‐effectiveness analysis of this randomized trial, in patients with isolated patellofemoral disease and when performed by experienced surgeons, PFA was less costly from a 1‐year hospital management perspective and was associated with more favorable short‐term outcomes compared with TKA [378]. In randomized comparisons of partial versus TKA, trials incorporating objective performance‐based tests—such as the 2‐min walk test and timed up‐and‐go—have shown that unicompartmental knee arthroplasty (UKA) is associated with faster functional recovery, reflected by earlier improvements in walking speed and sit‐to‐stand performance, compared with TKA [379], However, in another multicenter assessor‐blinded randomized trial with 2‐year follow‐up, despite faster early recovery with UKA, patient‐reported outcomes at 2 years were not superior to those of TKA [380]. Beyond conventional surgery, evidence for minimally invasive interventions targeting knee OA pain has also become more stringent. A RCT evaluating cryoradiofrequency ablation of periarticular knee nerves showed that the procedure produced significant analgesia beyond placebo effects; however, in the same study, functional status, medication use, and patient satisfaction did not differ between groups [381].

In hip OA, randomized evidence related to arthroplasty has focused in part on direct comparisons between total hip arthroplasty (THA) and hip resurfacing. Multicenter randomized trials have shown that, at 1‐year follow‐up, there was no evidence of a difference in hip function between the two procedures [382]. Earlier RCTs conducted in younger patients similarly focused on differences in functional outcomes at 1 year after surgery. These studies explicitly noted that, although hip resurfacing appeared to offer favorable short‐term functional prospects, it carried potential early risks—such as femoral neck fracture—thereby underscoring the need for RCTs to determine its true comparative effectiveness and safety relative to THA [383]. On the other hand, controlled studies comparing THA with education plus exercise programs have also been conducted. These trials consistently indicate that THA provides greater overall improvements in pain, function, and quality of life than education and exercise alone, supporting the clinical benefit of proceeding to arthroplasty in end‐stage hip OA [384].

## 7 Conclusion and Prospects

OA develops through the interplay of several interrelated factors and the breakdown of tissues throughout the joint, with the disorder arising from coordinated tissue remodeling processes involving four interconnected systems: (1) articular cartilage, (2) subchondral bone, (3) the synovium–IPFP, and (4) vascular and neural structures within the joint. These processes are driven by chronic inflammation, metabolic dysregulation, and abnormal joint loading. The epidemiology of OA indicates that population aging and the increasing prevalence of obesity and metabolic dysfunction are contributing to a growing disease burden and a more established chronic disease state. The association of OA with CVD, neurodegenerative disorders, and mental health conditions further suggests that the coordinated cellular responses (and pathological drivers) affecting the joint may also extend beyond the joint itself in the form of systemic inflammation, metabolic imbalance, and neuroimmune signaling.

With regard to clinical observations, pain does not always correspond to radiographic evidence of structural damage. In fact, patients may differ substantially in inflammatory activity, mechanical load imbalance, metabolic status, and response to therapy. These differences indicate that no single mechanistic model can adequately explain the complexity of OA across its diverse phenotypes; consequently, many traditional treatment modalities may work for some patients but fail to produce satisfactory outcomes in others in routine clinical practice.

The mechanisms by which inflammatory signaling, mechanotransduction, oxidative stress, and metabolic reprogramming interrelate will be assessed. Matrix degradation, chondrocyte phenotypic switching, cellular senescence, and various forms of programmed cell death are pathways that communicate through networks of crosstalk, including NF‐κB, MAPK, Wnt/β‐catenin, and PI3K–AKT–mTOR, and drive these processes, with consequences that include subchondral bone remodeling, increased synovial inflammation, and enhanced vascular and neural ingrowth. Consequently, these processes create a pathological cycle in which structural injury leads to acute inflammatory amplification and increased pain sensitivity. Rather than acting as independent switches, these pathways function as a coupled regulatory network; therefore, a single stimulus can trigger the parallel activation of multiple axes through converging transcriptional regulators, shared metabolic nodes, and cellular checkpoints governing developmental fate determination. As a result, single‐target interventions typically yield only short‐term or inconsistent structural benefits.

From this perspective, epigenetic changes such as DNA methylation, histone modifications, and ncRNAs correspond with the long‐term, gradual, cumulative nature of the problem. Epigenetically, the cellular experience of exposure to chronic inflammatory, mechanical, or metabolic stimuli can be documented and remain as a stable memory, affecting transcriptional programming, lowering the threshold to stress, and compromising homeostatic resilience. Thus, this perspective provides an explanation for the ongoing progression of joint disease in injured joints as well as the disparate outcomes observed in comparable individuals with joint injury.

At the therapeutic level, current clinical strategies remain centered on symptom control and functional improvement, reflecting both the chronic complexity of OA and the fact that truly structure‐modifying therapies are still in an exploratory phase. Nonpharmacological interventions form the foundation of long‐term management. Exercise training, weight management, education and self‐management programs, digital or remote health support, and modalities such as Tai Chi or yoga have consistently been shown to improve pain and function and enhance adherence, with added value for patients with metabolic disturbances or psychological stress. However, their effectiveness is highly dependent on sustained implementation, feasibility, and alignment with patient phenotypes; in real‐world settings, benefits may diminish with insufficient intensity, poor adherence, or severe malalignment. Pharmacological treatment is primarily analgesic and anti‐inflammatory in intent and requires careful balancing of efficacy against long‐term safety, particularly GI, cardiovascular, and renal risks. Its impact on structural degeneration remains limited. Intra‐articular therapies—including HA, selected biomaterials or viscosupplementation approaches, and tissue‐derived products—have shown symptomatic benefits and feasibility in some studies. Nevertheless, heterogeneity in product composition, manufacturing processes, indications, and outcome measures has led to inconsistent results, and robust long‐term evidence demonstrating true modification of structural disease progression is still lacking. The slow advance of DMOAD development is therefore driven less by a shortage of candidate agents than by a set of fundamental challenges: dilution of treatment effects due to OA heterogeneity; discordance between symptomatic and imaging‐based structural outcomes; long follow‐up periods and high trial costs; insufficient standardization of imaging quantification and biomarker endpoints; and the need to balance efficient local delivery with systemic safety.

Future OA research and therapy will likely transition toward a model characterized by phenotype‐driven stratification, combination therapies, and the use of quantifiable outcomes instead of the longstanding “one target–one patient” approach. Meaningful breakthroughs in OA will arise from establishing a systematic translational pathway that encompasses multiple targets rather than single targets. For example, diseases should no longer be categorized primarily by site or radiologic findings but rather by their underlying mechanisms. Clinically relevant phenotypes can largely be organized into categories such as inflammation‐dominant, mechanical‐dominant, subchondral bone remodeling‐dominant, and neurovascular reorganization‐dominant, either as distinct groups or along a continuum. The value of such stratification lies not in conceptual proliferation, but treatment matching: an inflammatory phenotype may be more responsive to anti‐inflammatory or immunomodulatory approaches; a metabolic phenotype requires targeting body weight, insulin resistance, adipokines, and mitochondrial metabolism; a mechanical phenotype should prioritize load redistribution via gait, muscle strengthening, alignment correction, bracing, and rehabilitation; and phenotypes characterized by prominent subchondral remodeling or neurovascular changes may require combined strategies focusing on the osteochondral unit and pain pathways.

As a second point, the rationale for combining different intervention strategies needs to be clearer. Nonpharmacological interventions should provide an ongoing and stable foundation to reduce the systemic and inflammatory risks associated with OA. On this basis, pharmacological or intra‐articular therapies can be layered according to clinical phenotype, while DMOADs with structural effects should be explored in combination with nonpharmacological interventions and/or in sequential regimens. To avoid a “one‐size‐fits‐all” strategy and develop adaptive treatment pathways dynamically optimized according to disease stage and individual clinical symptoms and structural changes, mechanistic research must align more closely with clinical reality. This includes focusing on defining the temporal roles of dysregulated pathways across the various tissues involved, disease stages, and cellular or subcellular populations, as well as determining whether modulation of these pathways should involve inhibition or restoration to a physiological range, thereby minimizing the rate of translational failure.

Frameworks for evaluation need greater standardization and reproducibility. The common reliance on either a single pain score or a radiographic grade alone is often insufficient to reflect structural progression or capture phenotypic heterogeneity. For a more pragmatic approach, multidimensional composite endpoints should be created that integrate symptoms (pain and function), structural measures (quantitative MRI metrics, volumetric bone marrow lesions, cartilage thickness, morphology, or composition), and inflammatory or biochemical indicators (biomarkers of inflammation, metabolism, and bone and cartilage turnover), with prespecified stratified statistical analyses embedded within the trial design. This methodology should enhance statistical efficiency while more accurately reflecting the chronic and heterogeneous nature of OA.

Ultimately, the potential for disease‐modifying therapies of conditions such as OA is promising but challenging. The success of implementation will depend on the degree of precision and foresight achieved in therapeutic design. A more realistic objective is to selectively modulate specific regulatory nodes within defined cell populations or disease stages while minimizing systemic effects through local delivery or targeted strategies. Meanwhile, epigenetic markers may serve more immediately as surrogate indicators or predictors of phenotypic variation and risk stratification rather than as universal therapeutic targets.

OA is generally characterized by chronic instability of joint homeostatic regulation and will benefit from multifaceted approaches that incorporate treatment modalities based on distinct pathogenic mechanisms into actionable combination frameworks; create new, standardized multidimensional outcomes to assist in the interpretation of clinical trials and determine the true effectiveness of therapies; translate DMOADs from a concept to clinical application with demonstrated structural improvement of the joint through restored homeostasis; and establish a new paradigm for the systemic treatment of OA. This paradigm more closely reflects the biology of OA and provides the most pragmatic path for transitioning from symptom control to modifying disease progression.

## Author Contributions

Tianrui Chen and Wenlong Chen conceived the scope and structure of the review and drafted the manuscript. Tianpeng Xu and Hong Wang participated in literature retrieval, critical analysis, and organization of the content. Yuheng Zhang and Li Wang contributed to data interpretation, figure conceptualization, and manuscript revision. Sijie Zhu assisted with literature curation and reference management. Huagiang Tao and Xing Yang provided overall supervision, critically revised the manuscript for important intellectual content, and approved the final version. All authors read and approved the final manuscript.

## Funding

This work was supported by the Jiangsu Provincial Department of Science and Technology (Grant No. BE2022737), the Suzhou Science and Technology Bureau (Grant No. SKYXD2022089), and the Nanjing Medical University Mingcheng Innovative Postdoctoral Research Program (Grant No. GSBSHKY202504).

## Ethics Statement

The authors have nothing to report.

## Conflicts of Interest

The authors declare no conflicts of interest.

## Data Availability

The authors have nothing to report.
